# Global identification of mRNA-interacting circular RNAs by CLiPPR-Seq

**DOI:** 10.1093/nar/gkae058

**Published:** 2024-02-07

**Authors:** Suman Singh, Sharmishtha Shyamal, Arundhati Das, Amaresh C Panda

**Affiliations:** Institute of Life Sciences, Nalco Square, Bhubaneswar, Odisha 751023, India; Regional Center for Biotechnology, Faridabad, Haryana 121001, India; Institute of Life Sciences, Nalco Square, Bhubaneswar, Odisha 751023, India; Institute of Life Sciences, Nalco Square, Bhubaneswar, Odisha 751023, India; Institute of Life Sciences, Nalco Square, Bhubaneswar, Odisha 751023, India

## Abstract

Although the functional role of circular RNA (circRNA) interaction with microRNAs and proteins has been studied extensively, circRNA interactions with the protein-coding mRNAs in intact cells remain largely unknown. Here, by employing AMT-mediated proximity ligation of RNA-RNA duplexes followed by circRNA enrichment and deep sequencing, we report a novel Cross-Linking Poly(A) Pulldown RNase R Sequencing (CLiPPR-seq) technology which identified hundreds of mRNA-interacting circRNAs in three different cell types, including βTC6, C2C12 and HeLa cells. Furthermore, CLiPP-seq without RNase R treatment was also performed to identify the mRNA expression in these cells. BLAST analysis of circRNAs in CLiPPR-seq sample with the mRNAs in CLiPP-seq samples determined their potential complementary sequences for circRNA-mRNA interaction. Pulldown of circRNAs and poly(A) RNAs confirmed the direct interaction of circRNAs with target mRNAs. Silencing of mRNA-interacting circRNAs led to the altered expression of target mRNAs in βTC6 cells, suggesting the role of direct interaction of circRNAs with mRNAs in gene expression regulation. CLiPPR-seq thus represents a novel method for illuminating the myriad of uncharacterized circRNA–mRNA hybrids that may regulate gene expression.

## Graphical Abstract

**Graphical Abstract:**
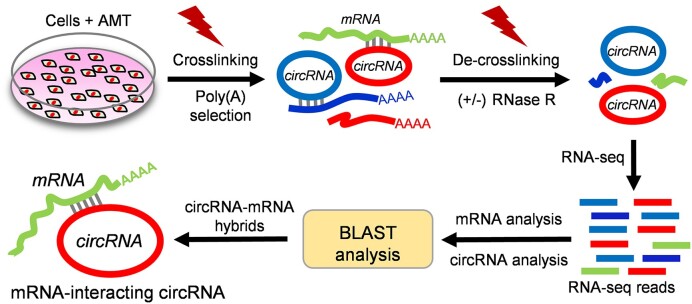

## Introduction

Although about 80% of the human genome is transcribed, <2% encodes protein (1). Therefore, classifying RNA into protein-coding and noncoding family has prevailed in biology for over 50 years (2). With accelerating sequencing techniques and advanced biochemical analysis tools, the existence and importance of noncoding RNA have surfaced significantly. Hundreds of noncoding RNAs have been functionally implicated in biological processes, but the mode of action or molecular mechanism behind those functions is difficult to characterize (3). The most widely used approach to understand the underlying mechanism is by deciphering the cellular components interacting with the noncoding RNAs. Most of the noncoding RNAs were discovered to interact with other RNAs either through protein or direct interaction, such as small nuclear RNA (snRNA)–mRNA, small nucleolar RNA (snoRNA)–rRNA/tRNA, long noncoding RNA (lncRNA)–microRNA (miRNA), miRNA–mRNA and circular RNA (circRNA)–miRNA (4–9). Since these RNA–RNA interactions (RRIs) are very critical for the regulation of gene expression, comprehensive mapping of these interactions could lead to a further understanding of their functions. Many RNA-centric biochemical methods conjugated with high-throughput sequencing techniques have emerged for identifying RNA–RNA interactions as well as predicting their structure, but with very fewer reports on circRNA interactions (4,5).

The genre of noncoding RNA comprises miRNA, tRNA, rRNA, lncRNA and most recently discovered circRNAs, which once were believed to be products of splicing errors (2). This noncoding class of circRNAs originates due to an unique backsplicing mechanism and has been extensively studied to understand its role in cellular complexities. Being serendipitously discovered in viruses, only a few studies undermined the potential impact of circRNAs until thousands of circRNAs were reported to be expressed from hundreds of human genes with the help of RNA-sequencing and specialized computational pipelines (10,11). With increasing focus on circRNA, multiple functions of circRNAs have been elucidated in the past decade, including its role as a protein scaffold, RNA Binding Proteins (RBP) sponge, modulator of rRNA maturation, transcription regulator, and the most widely studied function as miRNA sponge (9,12–14). Recent studies also report the association of circRNAs with translating ribosomes and suggest the translation of circRNAs into peptides through cap-independent translation mode (15).

Hundreds of studies revealed the circRNA association with either protein or miRNA to regulate cellular physiology (9,12). However, there has been no report on the transcriptome-wide analysis of the direct interaction of circRNAs with mRNAs. Given the versatile role of circRNA in development and diseases, the possibility of direct interaction of circRNA with mRNA was worth exploring. Therefore, we developed the CLiPPR-Seq, a sequencing technology coupled with proximity ligation and RNase R treatment to identify transcriptome-wide interaction of circRNAs with mRNA transcripts. Here, proximity ligation was primarily used to secure the RNA-RNA interactions, followed by poly(A) pulldown, ensuring a pool of mRNA and all its interacting counterparts. The specificity of these circRNAs was further determined by performing BLAST with mRNAs followed by analysis of circRNAs in mRNA pulldown and *vice versa* to prove the interactions. Furthermore, silencing of mRNA-interacting circRNAs led to changes in the expression of target mRNAs, suggesting a possible role of circRNA–mRNA interactions in gene expression. Together, we report an efficient technique to identify the novel unexplored circRNA–mRNA interactions that can provide new insight into posttranscriptional regulations.

## Materials and methods

### Cell culture and differentiation

Mouse βTC6 cells and C2C12 myoblast cells were cultured in Dulbecco's modified Eagle's medium (DMEM) supplemented with 15% fetal bovine serum (Gibco) and 1% penicillin-streptomycin (Thermo Fisher Scientific). For inducing differentiation into myotubes, sub-confluent C2C12 myoblast cells were switched to differentiation media comprising DMEM with 2% horse serum and 1% penicillin-streptomycin for 2 days. Human HeLa cells were cultured in DMEM medium supplemented with 10% fetal bovine serum (Gibco) and 1% penicillin-streptomycin.

### Overview of CLiPPR-Seq

*RNA–RNA proximity ligation:* The 4′-aminomethyltrioxsalen hydrochloride (AMT) stock solution of 1mg/ml was diluted in DMEM and 2× PBS for the working concentration of 20 μg/ml, which was used as irradiation media. The growth media of βTC6, 2-day differentiated C2C12 myotubes, and HeLa cells were replaced by the AMT media and incubated at 37°C for 30 minutes. As described earlier, UV crosslinking was performed at 365 nm on an ice-cool block for 7 min at a distance of 5 cm from the UV source for 2 cycles (16,17).

*Total RNA isolation:* RNA isolation was performed either by the conventional TRIzol reagent (Thermo Fisher Scientific) or with magnetic bead-based All RNA isolation kit (RNA Biotech) following manufacturer protocol. Briefly, βTC6, C2C12, and HeLa cells were lysed in RNA isolation reagent or TRIzol followed by precipitation of RNA in the aqueous layer or using the magnetic silica beads of All RNA isolation kit. RNA isolated by precipitation method was treated with DNase to remove DNA contamination. Quality and quantity of RNA were assessed using NanoDrop 2000 or Multiskan Sky and on Qubit4 (Thermo Scientific) using Qubit RNA High Sensitivity kit. Furthermore, RNA quality was analyzed on a TapeStation 4200 (Agilent) using an RNA ScreenTape or by resolving the RNA samples in a denaturing formaldehyde-agarose gel.

*Pulldown of poly(A) RNA:* 10 μg of total RNA isolated from AMT-treated samples was used for mRNA isolation using NEBNext® poly(A) mRNA Magnetic Isolation Module (NEB) as per manufacturer's instruction, and the quantity and quality of mRNA were assessed on Multiskan Sky (Thermo Scientific) and Qubit4 using Qubit RNA High Sensitivity kit.

*De-crosslinking and RNase R treatment:* For reverse-crosslinking, total mRNA along with its interacting counterparts pulled down from the βTC6, C2C12, and HeLa cell RNAs were irradiated at 254 nM for 5 min. Since exposure to 254 UV leads to degradation of RNA, we used 5 min of decrosslinking (18,19). De-crosslinked βTC6, C2C12, and HeLa RNA pool were subjected to RNase R treatment as previously described for enriching the interacting circRNA population (20).

*Library preparation and RNA-sequencing:* The cDNA libraries were prepared with the de-crosslinked poly(A) RNA with or without RNase R treatment. 25 ng of Poly(A) sample (CLiPP) and 10 ng of RNase R treated (CLiPPR) RNA samples from HeLa, C2C12, and βTC6 cells were used for library preparation using Collibri™ Stranded RNA Library Prep Kit (Thermo Fisher Scientific) following the manufacturer's instructions. The cDNA libraries were quality checked on a TapeStation 4200 (Agilent) using DNA ScreenTape, followed by 150 bp single-end sequencing on the Illumina NextSeq 550 platform.

### RNA sequencing analysis

Adaptor contamination was removed while converting the .bcl to fastq files using bcl2fastq (v2.20). Quality control was performed on the raw fastq files using Fastqc (v 0.11.8). More than 30 million reads were obtained in each sequencing samples and the clean fastq files were aligned to the mouse reference genome mm39 for βTC6 and C2C12 cells and human reference genome hg38 for HeLa cells. CircRNA annotation was performed using CIRCexplorer2 (v2.3.6) (21). For circRNA identification, the reads were aligned using the STAR aligner (v 2.7.1a) with the ChimSegmentMin-10 parameter of the CIRCexplorer2 pipeline (21). The aligned bed file (chimeric.out.junction) was used as an input to annotate the circRNAs (Supplementary Table S1-3). Raw data have been deposited in the European Nucleotide Archive (ENA accession Number is PRJEB58914). The mRNA annotation of the three CLiPP-seq libraries without RNase R treatment was performed using the STAR (v: 2.7.1a) aligner, and the raw counts were calculated using FeatureCounts from the Subread Package (v: 2.0.3). The mRNAs identified in these datasets were converted into nucleotide database using the –makedb parameter of Blast+ suite.

### Identifying conserved circRNA interaction sites

The current development in data science has established circRNAs to be conserved across several species (22,23). The HeLa (hg38) circRNA coordinates were converted to mouse mm39 circRNA coordinates using the LiftOver tool of the UCSC genome browser (24). The LiftOver circRNA coordinates of HeLa CLiPPR-seq data were compared with βTC6 and C2C12 CLiPPR-seq circRNA coordinates to identify conserved mRNA-interacting circRNAs (Supplementary Table S4).

### Detecting circRNA–mRNA pairs by BLAST+

The protein-coding transcripts with defined 5′ UTR, CDS, and 3′ UTR were considered for mRNAs database for the BLAST analysis. The sequences of circRNAs identified in CLiPPR-seq were used to blast against the mRNAs identified in the CLiPP-seq samples of the corresponding cell type. The customized minus strand .ASN file was created from RefSeq Select with the following additional criteria: somewhat similar sequences; max target sequence = 5000; threshold = 10; word size = 7; match/mismatch scores = 2, -3; gap costs = existence: 5, extension: 2. Further only Plus/Minus interacting results were selected to identify the interacting circRNA-mRNA hybrids. The specific regions of the interacting mRNAs, such as 5′ UTR, CDS and 3′ UTR were determined from the Gencode mRNA transcripts of mice (gencode.vM25.pc_transcripts.fa) for the βTC6 and C2C12 cells and human (gencode.v40.pc_transcripts.fa) for the HeLa cells. The location of circRNA interaction on the mRNA regions was determined from the above location information. The details of the circRNA–mRNA pairs from BLAST analysis can be found in Supplementary Tables S5–S7.

### RNA isolation, cDNA synthesis, RT-qPCR and Sanger sequencing

As described above, total RNA was isolated from the βTC6, HeLa and 2-day differentiated C2C12 cells. The cDNA was prepared with 1 μg of total RNA using the Maxima reverse transcriptase, High Capacity cDNA Reverse Transcription Kit (Thermo Fisher Scientific), or LunaScript RT SuperMix kit (NEB) following the manufacturer's instruction, followed by PCR amplification using specific primer sets (Supplementary Table S8). Using the circRNA-specific divergent primers, RT PCR was performed at 95°C for 2 minutes, followed by 40 cycles of 95°C for 5 s and 60°C for 20 s using 2× DreamTaq PCR master mix and resolved in SYBR gold stained 2% agarose gel. Additionally, the RT-PCR products were purified and subjected to Sanger sequencing with one of the divergent primers. Sanger sequencing results were analyzed to find the backsplice junction (BSJ) sequence of the target circRNAs. The RT-qPCR was conducted with QuantStudio real-time machine using 2× PowerUp SYBR Green qPCR master mix (Thermo Fisher Scientific). The qPCR cycling conditions and primers can be found in Supplementary Table S8. The relative RNA levels in poly-(A) pulldown, circRNA pulldown, and circRNA silencing experiments were normalized to *H2ac21* or *18s* rRNA.

### Poly(A) RNA and circRNA pulldown assay

For poly(A) RNA pulldown, βTC6, 2-day differentiated C2C12, and HeLa cells were treated with AMT, followed by crosslinking at 365 nm and total RNA isolation. 10 μg of total RNA with or without crosslinking was dissolved in RNA binding buffer, and Oligo d(T)25 beads were used for mRNA enrichment following the manufacturer's protocol (NEB). Then, the mRNAs were eluted from the oligo-dT beads in 20 μl of elution buffer, followed by de-crosslinking (only for crosslinked RNA pulldown) to segregate mRNA from its interacting RNAs. Finally, 12 μl of the de-crosslinked RNA sample and 1 μg of control total RNA were used for cDNA preparation with High-Capacity cDNA reverse transcription kit or Maxima reverse transcriptase enzyme followed by RT-qPCR analysis of selected circRNA targets.

The native circRNA pulldown using biotin-labeled antisense oligo (ASO) in βTC6 cells was performed as described previously, with some modifications (25). Briefly, βTC6 cells were treated with or without AMT, followed by crosslinking at 365 nm and washing the cells with cold PBS three times. The cells were then lysed with PEB supplemented with RNase and protease inhibitors, followed by incubation with 100 pmol biotin-labeled control ASO or ASO targeting the BSJ of target circRNAs for 1.5 h at 4°C. The lysates with biotin-labeled ASOs were then incubated with 50 μL streptavidin magnetic beads for 30 min at room temperature, followed by 3–4 washing with 1× TENT buffer and isolation of RNA from the magnetic beads. The RNA from AMT crosslinked samples were de-crosslinked at 254 nm UV. Both control and circRNA pulldown samples were subjected to cDNA synthesis and RT-qPCR analysis. Native pulldown of poly(A) mRNAs was also performed for both βTC6 and C2C12 cells using the oligo-dT beads following the above protocol described for circRNA pulldown (25).

To confirm the direct interaction of *circMtcl1* with target mRNA sequences, the circRNA-interacting sequences of *2310039H08Rik* and *Rps6kc1* mRNA along with 5 nt flanking sequences were cloned downstream of Renilla ORF in the psiCHECK2 vector. As a negative control mutant, we cloned the antisense sequences of the above control sequences of *2310039H08Rik* and *Rps6kc1* downstream of *Renilla* ORF in the psiCHECK2 vector. The βTC6 cells were transfected with psiCHECK2 vector containing circRNA target sequence or mutant sequence using Lipofectamin 3000 and incubated for 48 hrs, followed by ASO pulldown of *circMtcl1*. The pulldown RNA samples were subjected to cDNA synthesis and RT-qPCR analysis to check Renilla mRNA enrichment normalized to internal control firefly luciferase mRNA.

### CircRNA structure, miRNA target prediction and circRNA silencing

The structure of *circMtcl1* and *circAcbd3* were predicted using the RNAstructure web server (26). The bimolecular secondary structure prediction of the circRNA-mRNA hybrid was predicted using the circRNAs-mRNA interacting sequences plus 10 nucleotides flanking region sequences in the RNAstructure web server. The miRNAs targeting to *circMtcl1* and *circAcbd3* were predicted with the miRDB web tool, followed by identifying experimentally validated mRNA targets of circRNA-associated miRNAs reported in miRTarBase (27,28). The antisense GapmerR LNA oligos against the BSJ sequence of *circAcbd3* and *circMtcl1* were synthesized (Eurogentec) (Supplementary Table S8). The βTC6 cells were transfected with *circAcbd3* and *circMtcl1* LNAs at a concentration of 100 nM using Lipofectamine RNAiMAX for 2 days, followed by RNA isolation, cDNA synthesis, and RT-qPCR analysis of target RNAs.

### Statistical analyses and visualization

Student's t-test was used to calculate statistical significance for comparisons (*P < 0.05). All data in the figures were generated from at least three independent biological replicates, and the error bars represent means ± SEM in each figure. GraphPad Prism, R studio, Cytoscape software, or Microsoft Excel were used to generate the figures for data visualization.

## Results

### The CLiPPR-seq methodology

**Figure 1.**
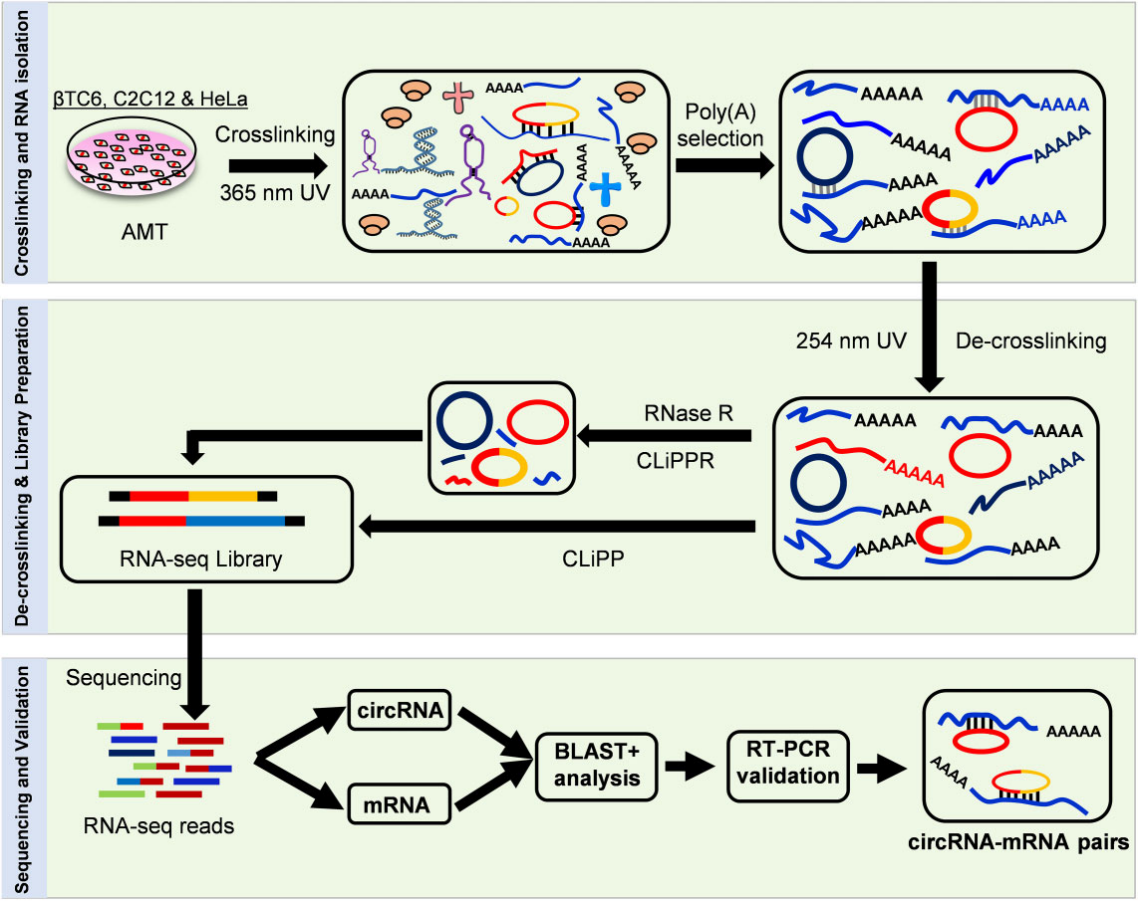
A step-by-step schematic representation of CLiPPR-Seq demonstrating the mapping of the circRNA-mRNA interactome by AMT crosslinking, poly(A) pulldown, RNase R treatment and sequencing to identify mRNA-interacting circRNAs.

Mouse pancreatic βTC6 cells were used to establish the CLiPPR-seq method to identify transcriptome-wide mRNA-circRNA interactome. As shown in Figure 1, CLiPPR-seq implements *in vivo* RNA duplex crosslinking using AMT, which, when irradiated at 365 nm can intercalate into the RNA duplex and crosslink RNA-RNA hybrids (29). Total RNA from βTC6 cells was extracted and used for affinity pulldown of poly(A) RNA using oligo-dT beads to identify circRNAs associated with mRNAs systematically. Further, crosslinking in the mRNA pulldown sample was reversed upon irradiation at 254 nm to separate all the mRNA interacting RNAs. Since mRNA pulldown can lead to the enrichment of mRNAs along with all mRNA-interacting non-poly(A) RNAs, RNase R treatment was performed on one-half of the sample for the enrichment of circRNAs followed by library preparation (CLiPPR-seq), whereas the other half of the sample was used for library preparation without RNase R treatment (CLiPP-seq) serving as mRNA library for identification of mRNA-interacting circRNAs. The CLiPP-seq samples served as a control or mRNA library for mapping the interacting circRNAs in the CLiPPR-seq samples. To prove the robustness of the CLiPPR-seq method, 2-day differentiated C2C12 myotubes, and HeLa cells were used to prepare CLiPP-seq and CLiPPR-seq libraries similar to βTC6 cells.

### CLiPPR-seq identified transcriptome-wide mRNA-interacting circRNAs

**Figure 2.**
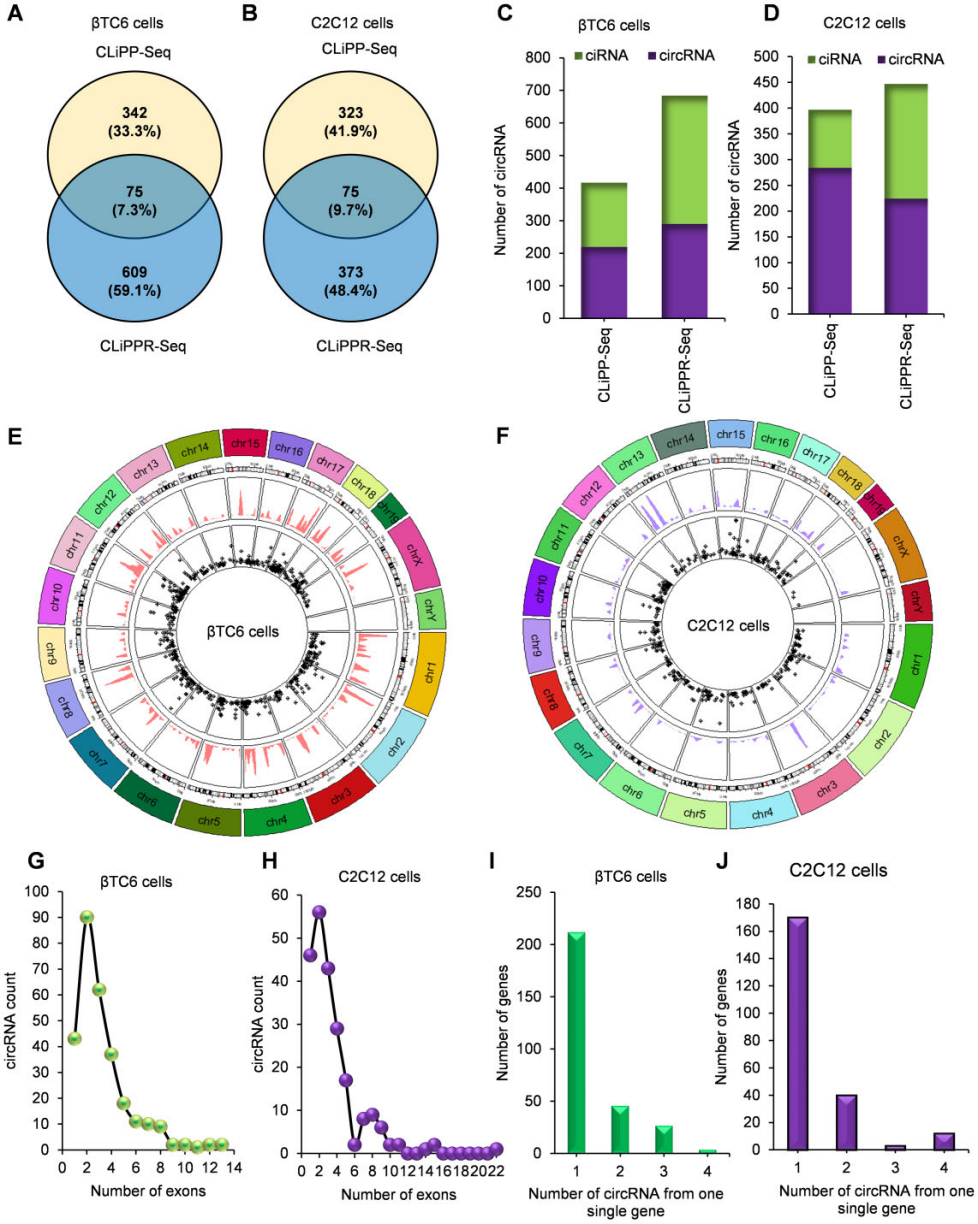
Characteristics of mRNA-interacting circRNAs in βTC6 and C2C12 cells. (**A**, **B**) CircRNAs identified by CIRCexplorer2 in βTC6 and C2C12 CLiPPR seq and CLiPP-seq samples. (C, D) The number of circRNAs that are generated from exonic (circRNA) or intronic (ciRNA) sequences in βTC6 (**C**) and C2C12 (**D**) cells. (E, F) Circos plot showing the distribution of circRNAs and their respective lengths along the chromosomal location in βTC6 (**E**) and C2C12 (**F**) cells. The internal circle with black stars shows the length distribution of the circRNAs and ciRNAs. (G, H) Dot plot showing the distribution of the number of exons in the circRNAs in βTC6 (**G**) and C2C12 (**H**) cells. (I, J) The bar graph represents the number of circRNAs generated per gene in βTC6 (**I**) and C2C12 (**J**) cells.

To identify the circRNA-mRNA interactome, the RNA-seq reads for all CLiPP-seq and CLiPPR-seq libraries were further processed for circRNA analysis. CIRCexplorer2 identified 1026 mRNA-interacting circRNAs in βTC6 CLiPPR-seq and CLiPP-seq samples. Interestingly, 684 mRNA interacting circRNAs were annotated in the βTC6 CLiPPR-seq, whereas 417 circRNAs were identified in βTC6 CLiPP-seq samples (Figure 2A), suggesting an enrichment of mRNA-interacting circRNAs with RNase R treatment. Out of the 1026 circRNAs, 75 circRNAs were common between the CLiPPR-seq and CLiPP-seq samples of βTC6 datasets (Figure 2A). Similarly, C2C12 CLiPPR-seq identified 448 mRNA-interacting circRNAs, whereas 398 were identified in CLiPP-seq with a fraction of circRNAs that are common between CLiPP and CLiPPR-seq (Figure 2B). Furthermore, CLiPP and CLiPPR-seq in HeLa cells identified 508 and 518 mRNA-interacting circRNAs, with more than half circRNAs common to both methods (Supplementary Figure S1A). CircRNA is known to be generated from both exonic and intronic sequences. Although the majority of the reported circRNAs are expected to be generated from exons, our data indicated that about 30% of mRNA-interacting circular RNAs were intronic (ciRNAs) in the CLiPP/CLiPPR-seq samples of βTC6, C2C12 and HeLa cells (Figure 2C, D, Supplementary Figure S1B). Since there was little overlap between CLiPP-seq and CLiPPR-seq samples, and some of the mRNA-interacting circRNAs identified in the CLiPP-seq library without RNase R treatment could be false-positive linear RNAs containing backsplice junction sequence, we only considered the CLiPPR-seq circRNAs for further analysis. Circos plot for all three datasets represents the length and distribution of circRNAs across the mouse and human chromosome cytobands (Figure 2E, F; Supplementary Figure S1C). Most of the circular RNAs identified in the CLiPPR-seq samples harbor less than ten exons in all the three βTC6, C2C12 and HeLa cells (Figure 2G, H; Supplementary Figure S1D). Although one to two circRNAs are generated from most of the host genes, a few of the host genes generate multiple circRNAs (Figure 2I, J, and Supplementary Figure S1E).

### Hundreds of circRNAs are predicted to interact with mRNAs in βTC6 and C2C12 cells

**Figure 3.**
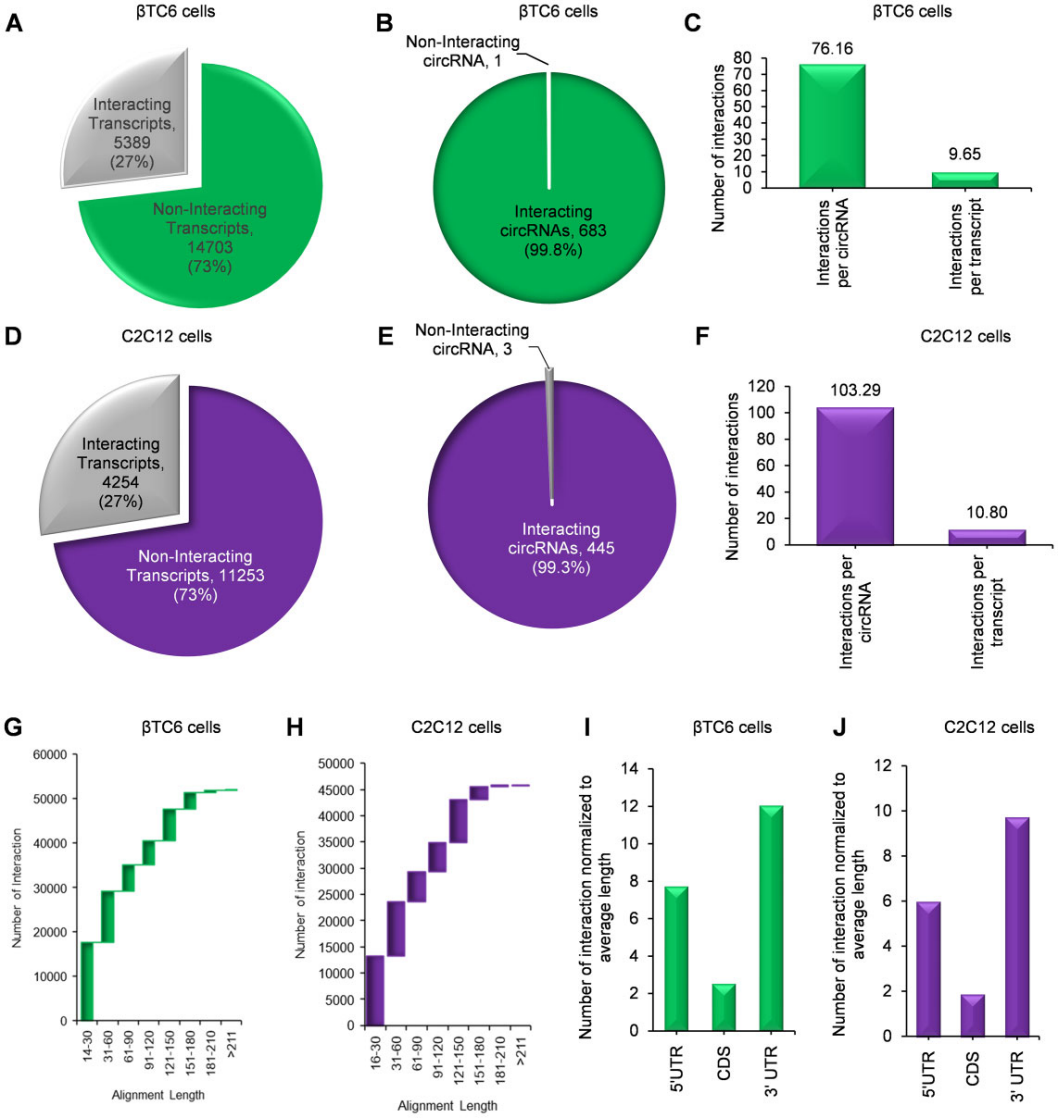
BLAST analysis of mRNA-interacting circRNAs in βTC6 and C2C12 cells.(A, B) The percentage of interacting mRNAs (**A**) and circRNAs (**B**) identified in the BLAST analysis in βTC6 cells. (**C**) Number of interactions per circRNA and mRNA in βTC6 cells. (D, E) BLAST analysis shows the percentage of interacting mRNAs (**D**) and circRNAs (**E**) in C2C12 cells. (**F**) Number of interactions per circRNA and mRNA in C2C12 cells. (G, H) Graphs showing the alignment length of circRNA–mRNA interactions in βTC6 (**G**) and C2C12 (**H**) cells. (I, J) Bar graphs depict the circRNA interactions in different regions of interacting mRNAs in βTC6 (**I**) and C2C12 (**J**) cells.

BLAST analysis was performed between the circRNAs identified in CLiPPR-seq against the custom mRNA nucleotide database of CLiPP-seq samples of the same cells to identify all the mRNA-circRNA interactions. With a minimum complementarity of 14 bp, the minus strand short sequence BLAST identified about 52,000 mRNA-circRNA hybrids in βTC6 cells (Supplementary Table S5). BLAST analysis found that about 27% of the mRNAs in CLiPP-seq have complementary sequences with the CLiPPR-seq circRNAs in βTC6 cells (Figure 3A). Similarly, BLAST analysis discovered that around 50% of the pancreatic islet circRNAs reported by PanCircBase interact with mRNAs identified in βTC6 CLiPP-seq, suggesting that only a small fraction of all expressed circRNA have sequence complementarity with mRNAs by chance (Supplementary Figure S2) (30). Importantly, >99% of circRNAs identified in βTC6 cell CLiPPR-seq data were found to have sequence complementarity with mRNAs identified in CLiPP-seq, indicating a specific enrichment and identification of mRNA-interacting circRNAs in βTC6 cells with CLiPPR-seq (Figure 3B). The circRNA BLAST with the mRNA transcripts in CLiPP-seq identified about 76 interactions per circRNA and 9 interactions per mRNA in βTC6 cells (Figure 3C). Similarly, BLAST analysis of circRNAs identified in C2C12 CLiPPR-seq with mRNAs in CLiPP-seq datasets revealed that more than 99% of circRNAs in CLiPPR-seq have sequence complementarity with 27% of mRNAs (Figure 3D, E). Moreover, BLAST analysis revealed >45,000 circRNA-mRNA pairs in C2C12 cells, with an average of about 103 interactions per circRNA and 10 interactions per mRNA (Figure 3F, Supplementary Table S6). BLAST analysis of HeLa CLiPPR-seq circRNAs also showed similar results, wherein more than 99% of the circRNAs identified in HeLa cell CLiPPR-seq samples were predicted to interact with 26% of HeLa cell mRNAs identified in CLiPP-seq (Supplementary Figure S3A, B; Supplementary Table S7). Furthermore, BLAST analysis identified more than 431000 circRNA-mRNA interacting pairs in the HeLa dataset, with about 838 interactions per circRNA and about 96 interactions per mRNA (Supplementary Figure S3C).

Although the analysis of the circRNA–mRNA interaction alignment lengths suggested that most interactions were less than 60 base pair sequence complementarity between the circRNA and mRNAs in all three βTC6, C2C12 and HeLa cell datasets, there were hundreds of interacting pairs with >100 bp sequence complementarity (Figure 3G, H; Supplementary Figure S3D). Furthermore, we wanted to check the location of the circRNA-mRNA interactions on the mRNAs. The average length of 3′ UTR was found to be longer than the 5′ UTR and CDS of the mRNAs in all three CLiPP-seq datasets (Supplementary Figure S4A–C). To find the preferential interaction of circRNAs with specific regions of mRNAs, we normalized the number of circRNA–mRNA interactions with the average length of interacting mRNA regions. Interestingly, a significantly high number of normalized circRNA-mRNA interactions were found in the 5′ UTR and 3′ UTR compared to the CDS, suggesting the possible regulatory role of circRNA-mRNA interactions in βTC6, C2C12 and HeLa cells (Figure 3I, J; Supplementary Figure S4D).

### Pervasive circRNA–mRNA interactions across different cell types

**Figure 4.**
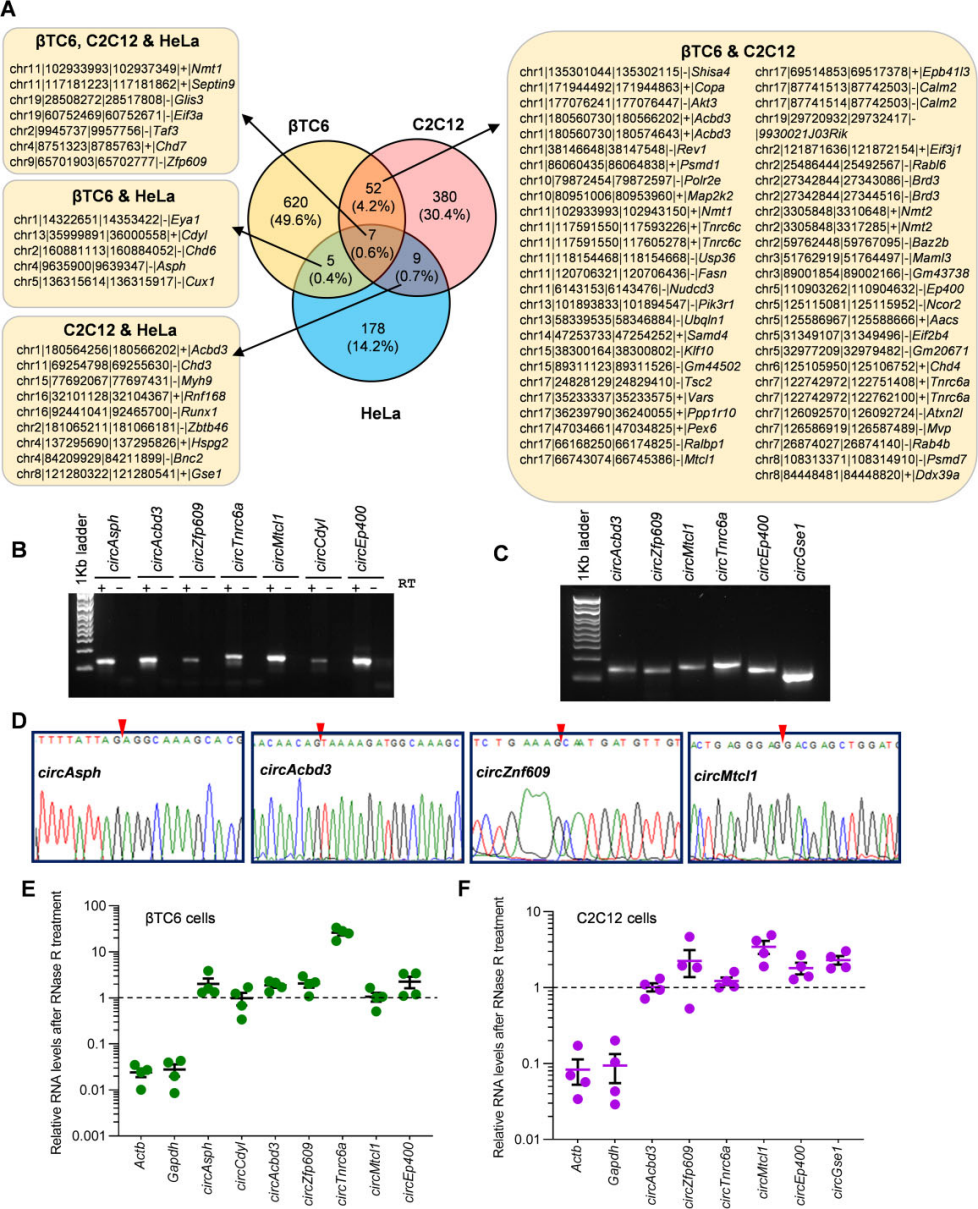
Validation of circRNA interacting with the mRNA transcripts.(**A**) Venny compares the mRNA-interacting circRNAs identified in CLiPPR-seq datasets of βTC6, C2C12 and HeLa cells. All the chromosomal IDs represent mouse mm39 coordinates. (B, C) Visualization of RT-PCR products of circRNAs in βTC6 (**B**) and C2C12 (**C**) cells in a 2% agarose gel stained with SYBR Gold. (**D**) Sanger sequencing of βTC6 circRNA products shows the circRNA junction sequence matching RNA-seq data. The red arrow depicts the BSJ sequence. (E, F) RT-qPCR analysis showing the levels of mRNAs and circRNAs upon RNase R treatment in βTC6 (**E**) and C2C12 (**F**) cells showing circRNA stability. The error bars in panels E and F represent means ± SEM from four independent experiments.

Several reports have reported the conservation of circRNA between humans and mice (22,31). LiftOver conversion of the human HeLa mRNA-interacting circRNA coordinates to Mouse coordinates discovered that 73 mRNA-interacting circRNAs are common in datasets from at least two cell types (Supplementary Table S4). We found 59 common circRNAs between βTC6 and C2C12 datasets, whereas 16 circRNAs were common between C2C12 and HeLa, and 12 circRNAs were common between βTC6 and HeLa datasets (Figure 4A). Only 7 circRNAs were found to be common among all the three βTC6, C2C12, and HeLa cells datasets (Figure 4A). Considering the abundance and length of common mRNA-interacting circRNAs, we selected a subset of circRNAs, including *circAsph*, *circAcbd3, circZfp609, circTnrc6a, circMtcl1, circCdyl, circEp400* and *circGse1* for validation in βTC6 and C2C12 cells, whereas *circASPH, circCDYL, circGSE1* were selected for validation in HeLa cells (Figure 4A; Supplementary Figure S1E). Since circRNAs show cell/tissue-specific expression patterns and the three cell lines used in this study are very different, we could find a few common circRNAs in these data sets (10,22,32). However, the identification of hundreds of mRNA-associated circRNAs in diverse cell lines indicates the widespread existence of novel circRNA–mRNA interactions, which needs further investigation.

### Validation of mRNA-interacting circRNAs

As shown in Figure 4B and C, RT-PCR was performed using divergent primer pairs to confirm the expression of the mRNA-interacting circRNAs in mouse βTC6 and C2C12 cells. Similarly, RT-PCR using divergent primers amplified the target circRNAs in human HeLa cells (Supplementary Figure S5A). Additionally, purification of the amplified PCR products followed by Sanger sequencing confirmed the specific amplification of the backsplice junction sequences of the target circRNAs (Figure 4D, Supplementary Figure S5B–D). As circRNAs are known to be resistant to RNase R treatment owing to their circular characteristics, we also subjected the total RNA from βTC6, C2C12 and HeLa cells to RNase R treatment, followed by the analysis of target linear and circRNAs. As expected, the linear *Actb* and *Gapdh* mRNAs were degraded, while the target mRNA-interacting circRNAs in βTC6 and C2C12 cells were resistant to RNase R digestion, suggesting their circular nature (Figure 4E, F). Similarly, the mRNA-interacting circRNAs in Hela were resistant to RNase R treatment, while the linear *ACTB* and *GAPDH* mRNAs were degraded by RNase R, confirming the circular nature of selected circRNAs in HeLa cells (Supplementary Figure S5E).

### CircRNAs are associated with mRNAs *in vivo*

**Figure 5.**
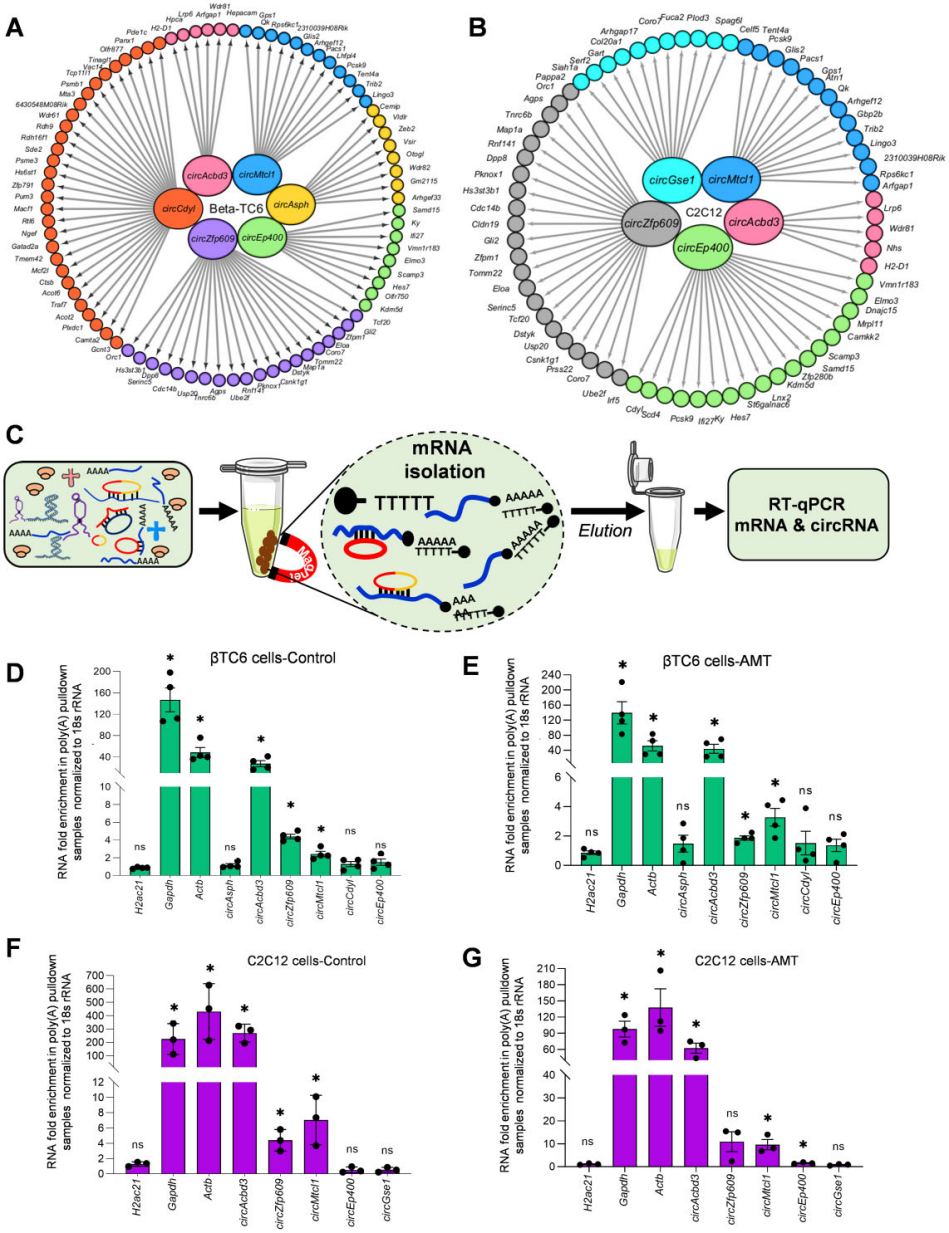
Enrichment of the mRNA interacting circRNAs in poly(A) pulldown sample. (A, B) Cytoscape image showing the mRNAs interacting with a subset of abundant circRNAs selected for validation in βTC6 (**A**) and C2C2 (**B**) cells. (**C**) A schematic representation of poly(A) pulldown and enrichment of mRNAs and mRNA-interacting circRNAs. (D, E) RT-qPCR analysis showing the enrichment of mRNAs and circRNAs in poly(A) pulldown samples compared to input using control total RNA (**D**) and AMT-crosslinked total RNA (**E**) of βTC6 cells. (F, G) RT-qPCR analysis showing the enrichment of mRNAs and circRNAs in poly(A) pulldown samples compared to input using control total RNA (**F**) and AMT-crosslinked total RNA (**G**) of 2-day differentiated C2C12 cells. The error bars in panel D–G represent means ± SEM from 3–4 independent experiments, and * indicates the statistical significance with a *P*-value <0.05.

We analyzed the BLAST data further to find the actual interaction of circRNAs with specific mRNAs. The BLAST results analysis identified several mRNAs interacting with validated circRNAs in βTC6, C2C12, and HeLa cells (Figure 5A, B; Supplementary Figure S6A). To validate the interaction of selected mRNA-interacting circRNAs with mRNAs, we performed poly(A) RNA pulldown assay with oligo-dT beads using total RNA from βTC6, and 2-day differentiated C2C12 myotubes, followed by the analysis of enriched circRNA/mRNAs (Figure 5C). Interestingly, several circRNAs, including *circAcbd3, circZnf609* and *circMtcl1*, were consistently enriched in the control βTC6 cell poly(A) RNAs as well as in AMT crosslinked βTC6 cell poly(A) RNAs compared to the input samples (Figure 5D, E). The enrichment of housekeeping *Gapdh* and *Actb* mRNAs, without the enrichment of non-poly(A) *H2ac21* mRNA in the pulldown samples indicates the specific pulldown of poly(A) RNAs and their interacting circRNAs. Similarly, poly(A) RNA pulldown from total RNA of HeLa cells followed by RT-qPCR suggested a specific enrichment of mRNA-interacting circRNAs including *circASPH, circCDYL* and *circGSE1* (Supplementary Figure S6B). Since poly(A) pulldown assay may identify false-positive circRNAs in the total RNA samples due to sequence complementarity, we performed poly(A) pulldown assay with the oligo-dT beads in the native βTC6 cell lysates. Interestingly, poly(A) pulldown of βTC6 cell lysates in native condition followed by enrichment analysis of mRNA-interacting circRNAs revealed that *circAcbd3, circMtcl1, circZnf609, circCdyl* and *circEp400* were enriched in poly(A) samples, confirming the possible *in vivo* interaction of these circRNAs with mRNAs in βTC6 cells (Supplementary Figure S6C). Similarly, *circAcbd3, circZnf609* and *circMtcl1* were enriched in the control C2C12 cell poly(A) RNAs, while *circAcbd3 and circMtcl1* were significantly enriched in AMT crosslinked C2C12 cell poly(A) RNA samples compared to the input (Figure 5F,G). In addition, *circAcbd3* was significantly enriched in the poly(A) pulldown samples using 2-day differentiated C2C12 cell lysates, indicating the consistent interaction of circRNAs with mRNAs *in vivo* (Supplementary Figure S6D). Moreover, the enrichment of *circZnf609* in poly(A) samples supports the interaction of *circZnf609* with target mRNA, which was reported recently (33).

As a proof-of-principle, a few potentially interacting mRNAs were selected for analyzing their enrichment in the same poly(A) samples, where we detected enriched mRNA-interacting circRNAs. As expected, RT-qPCR analysis on mRNAs predicted in BLAST to interact with the validated circRNAs were enriched in the βTC6 and C2C12 poly(A) samples, suggesting that the mRNA-interacting circRNAs could be enriched in the poly(A) RNA pulldown samples due to their association with target mRNAs *in vivo* (Supplementary Figure S6E, F). Interestingly, *circAcbd3*, being the most enriched circRNA in the poly(A) pulldown samples, was found to have sequence complementarity with many mRNAs, among which *Arfgap1, Hpca*, and *H2-D1* were enriched in βTC6 cells, while *Arfgap1 mRNA* was enriched in C2C12 cells (Supplementary Figure S6E,F). Similarly, *circMtcl1*, having complementarity with *Trib2, Rps6kc1* and *2310039H08Rik*, were enriched in the poly(A) pulldown in βTC6 and C2C12 cells. Among the other circRNAs, a few mRNA targets of *circAsph* and *circZnf60*9 were also enriched in the respective cell lines (Supplementary Figure S6E, F). The consistent enrichment of *circAcbd3 and circMtcl1* in the poly(A) pulldown samples indicated their possible direct interaction with target mRNAs in cells.

### CircRNAs directly interact with target mRNAs based on sequence complementarity

**Figure 6.**
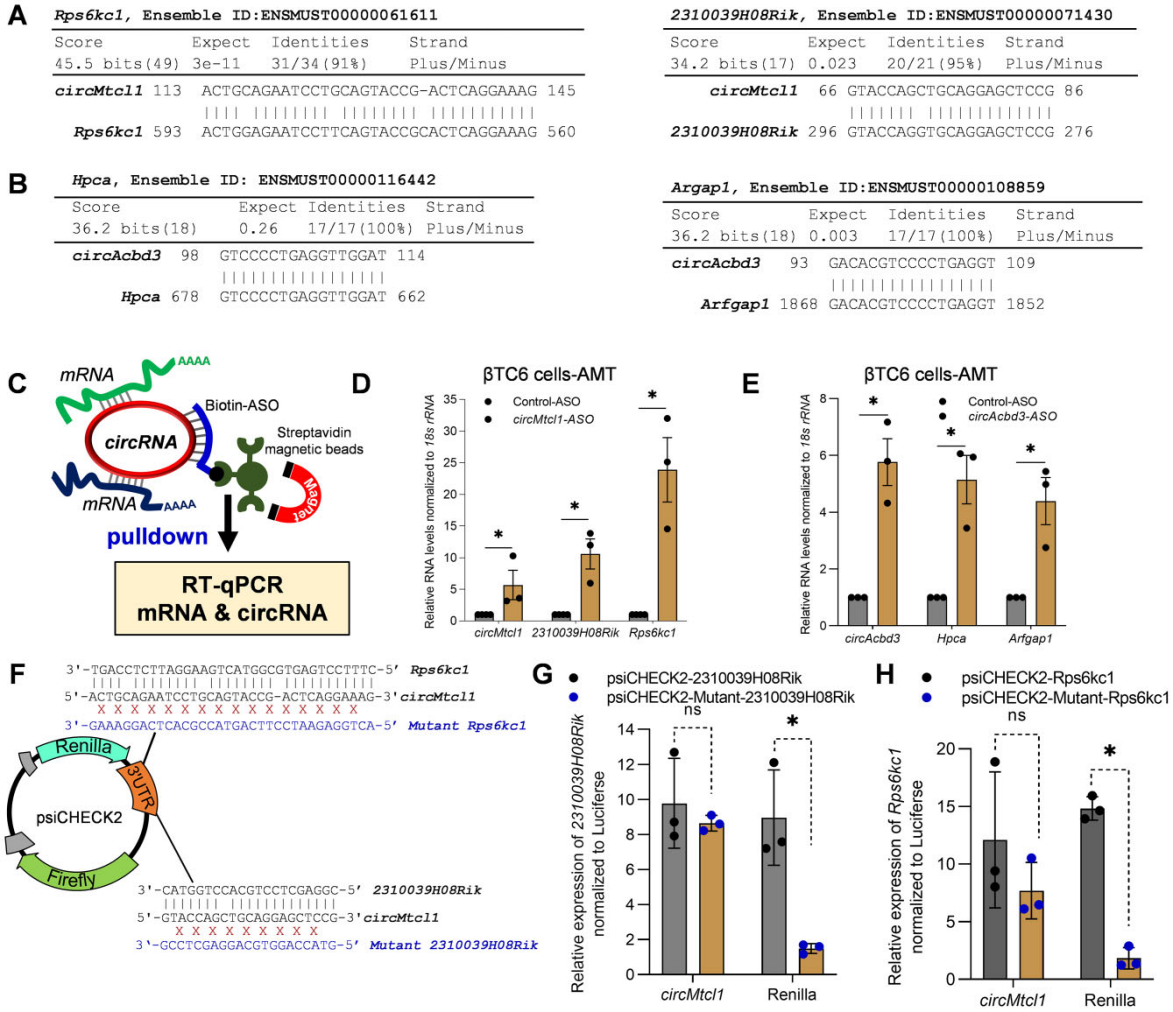
Validation of direct interaction of circRNA with target mRNAs. (**A**) BLAST results showing sequence complementarity of *circMtcl1* with *2310039H08Rik* and *Rps6kc1* mRNA. (**B**) BLAST analysis predicting the sequence complementarity of *Hpca* and *Arfgap1* mRNAs with *circAcbd3*. (**C**) Schematic representation of circRNA pulldown strategy using biotin-labeled ASO in AMT-treated βTC6 cells. (D, E) RT-qPCR analysis showing the enrichment of target mRNAs in *circMtcl1* (**D**) and *circAcbd3* (**E**) ASO pulldown samples compared to control ASO in AMT crosslinked βTC6 cells. (**F**) Schematic representation of psiCHECK2 cones with target and mutant sequences of mentioned mRNA sequences targeted by *circMtcl1*. (**G**, **H**). RT-qPCR analysis showing the enrichment of targets in *circMtcl1-*ASO pulldown samples of βTC6 cells transfected for 2 days with the mentioned psiCHECK2 target or mutant vectors. The error bars in panels D, E, G, H represent means ± SEM from 3–4 independent experiments and * indicates the statistical significance with *P*-value <0.05.

As shown in Figure 6A, B, BLAST analysis suggested that *circMtcl1* has complementarity with *2310039H08Rik* and *Rps6kc1* mRNA, while *circAcbd3* has sequence complementarity with *Arfgap1* and *Hpca* mRNA. As internal base-pairing in the circRNA sequence may hinder the interaction of circRNA with target mRNA sequences, we analyzed the secondary structure of *circAcbd3* and *circMtcl1* with their target mRNA sequences using the RNAstructure web server (26). As shown in Supplementary Figure S7, the prediction of bimolecular secondary structure *circMtcl1* using the RNAstructure web server predicted a long stretch of *circMtcl1* base-pairing with *2310039H08Rik* & *Rps6kc1*, while the interacting region of *circMtcl1*have small stretches of internal base-pairing which may not affect circRNA-mRNA interaction. Similarly, the prediction of biomolecular secondary structures of *circAcbd3* interaction with *Arfgap1* and *Hpca* mRNA sequences indicated a long stretch of circRNA-mRNA base-pairing, while the internal base-pairing in the interacting region of *circAcbd3* shows small stretches of double-stranded regions (Supplementary Figure S8). Together, the bimolecular secondary structure prediction and interaction data suggest that the interactions of circRNA–mRNA found by BLAST analysis of circRNAs in CLiPPR-seq may not be hindered by the internal base-pairing and secondary structures of circRNAs, at least for the validated circRNA–mRNA pairs.

To find the direct interaction of circRNA-mRNA pairs *in vivo*, βTC6 cells were treated with AMT, followed by crosslinking to stabilize the mRNA-circRNA interactions. The pulldown of *circAcbd3* and *circMtcl1* with ASO targeting the BSJ showed significant enrichment of these circRNAs along with their target mRNAs in βTC6 cells, suggesting a direct interaction of circRNA and target mRNAs in native cellular conditions due to sequence complementarity (Figure 6C–E). In parallel, the pulldown of *circAcbd3* and *circMtcl1* with ASO in βTC6 cells without AMT crosslinking enriched the circRNAs and target mRNAs (Supplementary Figure S9A, B). To further prove the direct interaction of circRNAs with target mRNA sequences, we cloned the *circMtcl1*-interacting mRNA sequences and their mutant (reverse complementary sequence) downstream to the Renilla ORF in psiCHECK2 vector (Figure 6F). The control and mutant plasmids were transfected into βTC6 cells for 2 days, followed by enrichment analysis of target circRNAs and *Renilla* mRNA in *circMtcl1* ASO pulldown samples. Importantly, the *Renilla* mRNA 3′ UTR clones with target mRNA sequences of *2310039H08Rik* and *Rps6kc1* were significantly enriched compared to the mutant in the *circMtcl1*ASO pulldown in βTC6 cells, indicating specific direct interaction of *circMtcl1* with their target mRNA sequences *in vivo* (Figure 6G, H). CLiPPR-seq, BLAST analysis, poly(A) pulldown, and circRNA pulldown experiments suggest the direct interaction of circRNAs with mRNAs based on sequence complementarity.

### Regulation of mRNA expression by mRNA-interacting circRNAs

**Figure 7.**
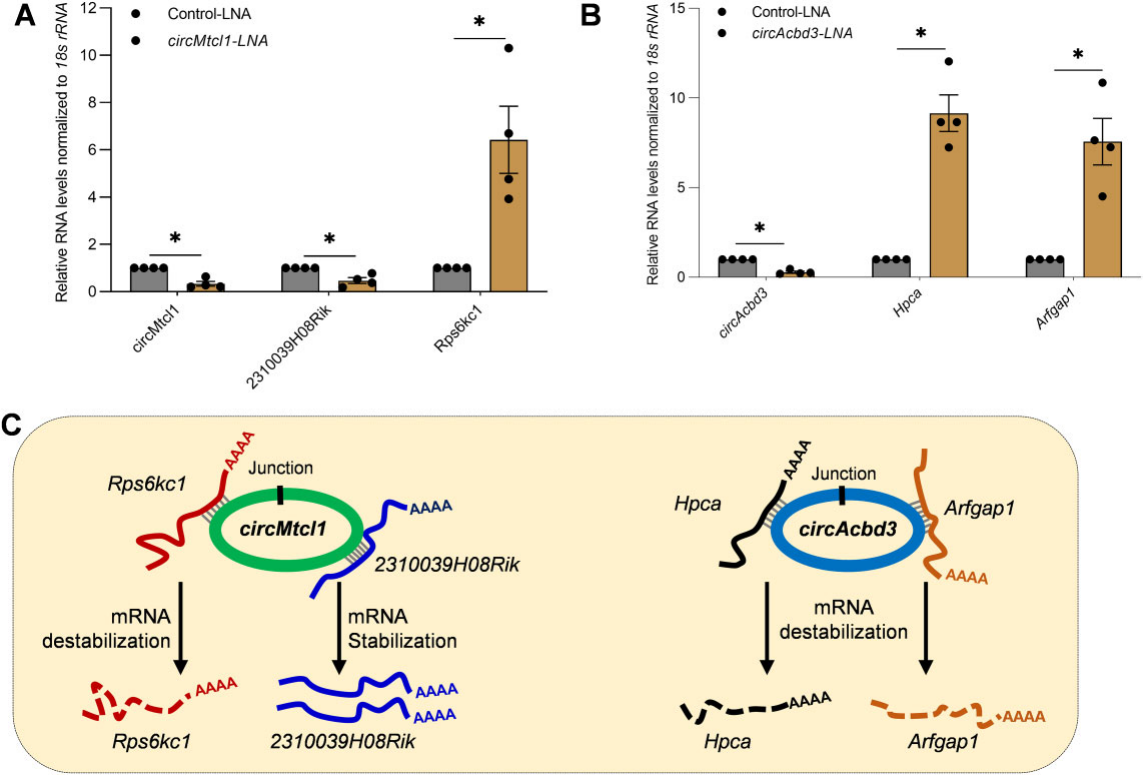
Regulation of mRNA expression by mRNA-interacting circRNA. (A, B) RT-qPCR analysis of interacting mRNAs upon silencing *circMtcl1* (**A**) and *circAcbd3* (**B**) in βTC6 cells. (**C**) Schematic representation of circRNA-mediated regulation of its interacting mRNAs through unknown mechanisms. The error bars in panels A and B represent means ± SEM from four independent experiments, and * indicates the statistical significance with a *P*-value <0.05.

To establish the direct effect of circRNA–mRNA interactions on target mRNA expression, we selected the consistently validated mRNA-interacting circRNAs, *circAcbd3* and *circMtcl1*, for silencing experiments. Interestingly, silencing *circMtcl1* in βTC6 cells led to a significant downregulation of *2310039H08Rik* and upregulation of *Rps6kc1* mRNA compared to the control GapmeR transfected cells (Figure 7A). Similarly, silencing *circAcbd3* led to a significant upregulation of the target *Arfgap1* and *Hpca* mRNAs (Figure 7B). To rule out the possibility of target gene expression through circRNA-miRNA axis, we predicted the miRNAs associated with *circMtcl1* and *circAcbd3* using miRDB, followed by identification of experimentally validated mRNA targets of those miRNAs using miRTarBase (Supplementary Figure S10A, B) (27,28). Interestingly, the validated *2310039H08Rik* and *Rps6kc1* mRNAs associated with *circMtcl1* were not targeted by the *circMtcl1*-associated miRNAs in βTC6 cells (Supplementary Figure S10C). Similarly, we could not identify a common target, including the validated *Arfgap1* and *Hpca* mRNAs, that are directly interacting with *circAcbd3*, and were targets of *circAcbd3*-associated miRNAs predicted by miRTarBase in βTC6 cells (Supplementary Figure S10D). Given that the mRNA expressions are altered upon *circAcbd3* and *circMtcl1* silencing without the possible involvement of miRNA-mediated regulation, our data indicate that the interaction of circRNA with target mRNA may lead to stabilization/destabilization of target mRNAs (Figure 7C). However, the association of the double-stranded region of circRNA–mRNA pair with different RBPs and their effect on the mRNA stability or translation cannot be ruled out. Although addressing the possible mechanism of target mRNA regulation by circRNA interaction needs to be explored in detail, the findings of this study open a novel avenue guiding future discoveries in circRNA research.

## Discussion

In recent years, high-throughput sequencing technologies revealed significant information on the vast diversity of the coding and noncoding transcriptome (2). RNA molecules function by interacting with RNA/protein. Notably, RNAs interact with self or other RNAs through base-pairing to regulate every step of posttranscriptional gene regulation, including pre-mRNA splicing, mRNA stability, and translation (4,5,17,34). For example, snRNA interaction with pre-mRNA regulates splicing, lncRNA/miRNA interaction with mRNA controls the translation or stability of mRNAs (4,5). Understanding the molecular RNA–RNA interactions (RRI) is the key to understanding their function. Several high and low-throughput methods have been developed to identify and characterize RRIs (4).

High-throughput identification of RRIs started with the development of cross-linking, ligation, and sequencing of hybrids (CLASH) technology that identified RRIs in yeast (35). Various methods have recently been developed to study physiologically relevant RRIs in various organisms, including humans (4). Since RBPs mediate several RNA-RNA interactions, several protein-centric high-throughput methods like CLASH, MARIO, RIC-seq, AGO-CLIP, hiCLIP and PIP-seq have enabled the capture of the structure and function of the intra- and intermolecular interactions (35–40). Similarly, RNA-centric methods such as PARS, PARTE, FragSeq, SHAPE-seq, LIGR-seq, PARIS and SPLASH have been developed to identify transcriptome-wide RNA–RNA hybrids (41–46). All these methods used crosslinking agents to stabilize the RNA hybrids, followed by digestion of single-stranded RNAs, proximity ligation of the RNA ends, and sequencing to identify intra- and intermolecular RNA–RNA interactions. However, although thousands of RNA–RNA interaction pairs have been discovered, the complete map of RNA–RNA interactome is far from complete due to the limitations associated with these methods and the lack of knowledge on novel RNA molecules like circRNAs.

CircRNAs are increasingly recognized as novel regulators of gene expression by controlling transcription, splicing, mRNA translation, and protein functions (9,12–14). Notably, all these functions of circRNA are mediated by its interaction with target RNAs and proteins. Hundreds of studies have highlighted the role of miRNA interaction with circRNAs or lncRNAs to regulate downstream genes through competitive endogenous RNA (ceRNA) networks (9). In addition, various studies have demonstrated the direct interaction of circRNA and miRNA, suggesting their importance in normal physiology and disease conditions. A recent study demonstrated that *circZNF609* regulates microtubule dynamics and tumorigenesis by directly interacting with *CKAP5* mRNA (33). However, the transcriptome-wide map of circRNA-mRNA interaction has not been studied in any cell type.

This study used AMT to crosslink the nearby uridines in the RNA-duplexes by irradiating at 365 nm UV (19,47). The crosslinking of RNA duplexes in the cell makes it possible to isolate all mRNA-interacting RNA by pulling down poly(A) RNAs with oligo-dT beads. The poly(A) RNAs and their associated RNAs were reverse crosslinked at 254 nm UV followed by digestion with RNase R to enrich the circRNA population (19). Since exposure to 254 UV leads to RNA degradation, exposure time was limited to 5 minutes (18,47). The reverse crosslinked poly(A) RNA (CLiPP) and RNase R digested poly(A) RNA (CLiPPR) samples were sequenced to identify all the mRNA-interacting circRNAs *in vivo* (Figure 1). Surprisingly, we detected hundreds of mRNA-interacting circRNAs in βTC6, C2C12 and HeLa cells. As expected, CLiPPR-seq identified a significantly higher number of circRNAs than CLiPP-seq in βTC6 cells due to the enrichment of circRNAs with RNase R treatment. Since a few circRNAs were common between CLiPP-seq and CLiPPR-seq samples, and RNase R treatment enhances the possibility of identifying true circRNAs, we only considered circRNAs identified in CLiPPR-seq samples for further validation. To ensure reliability, BLAST analysis was performed between the circRNAs identified in CLiPPR-seq against the mRNAs detected in the CLiPP-seq datasets of respective cells. Notably, most circRNAs detected here had multiple sequence complementarity with the UTR regions of mRNAs, suggesting a specific regulatory significance of circRNA–mRNA interactions (Figure 3). Furthermore, the enrichment of mRNA-interacting circRNAs in the poly(A) pulldown sample confirmed the *in vivo* interaction of circRNAs with mRNAs (Figure 5). Although BLAST analysis discovered complementary sequence matches for most of the circRNAs identified in CLiPPR-seq data, a small percentage of them did not have complementarity with mRNAs. The mRNAs without sequence complementarity with mRNAs could be enriched in the poly(A) samples due to indirect association with mRNAs through other RNA molecules. In addition, we could identify 21 circRNAs with at least 20 As at a stretch in βTC6 cells CLiPPR-seq data suggesting that these circRNAs could be enriched directly through interaction with oligo-dT beads or with poly(A) mRNAs. Similarly, we could find 15 and 56 circRNA with at least 20 As at a stretch in C2C12 and HeLa CLiPPR-seq data sets. However, BLAST analysis of these circRNAs with long poly(A) stretches was also predicted to have sequence complementarity with mRNAs, suggesting their possible interaction with mRNAs. In addition, to further validate the interaction of circRNAs with mRNAs, we performed an mRNA pulldown assay followed by the analysis of mRNA-interacting circRNAs as well as circRNA pulldown assay followed by the analysis of target mRNA enrichment (Figure 5,6). Interestingly, a subset of circRNA–mRNA interactions were validated in βTC6 and C2C12 cells.

Together, we demonstrate the utility of CLiPPR-seq that enables the transcriptome-wide identification of mRNA-interacting circRNAs. CLiPPR-seq complements recently developed high-throughput methods detecting global RNA-RNA interactions and reveals previously inaccessible information on circRNA–mRNA interactions that advise further studies on novel circRNA functions. Moreover, CLiPPR-seq can be applied in comparative studies of various conditions to find the altered circRNA–mRNA interactions that govern various pathophysiological phenomena. These findings thus help guide the functional analysis of unexpected circRNA–mRNA interactions, especially their role in regulating mRNA localization, stability, or translation. As shown in Figure 7, silencing of *circAcbd3* and *circMtcl1* altered the expression of target mRNAs, suggesting a new mechanism of circRNA-mediated gene regulation. It might be possible that the interaction of circRNA with target mRNA leads to displacement of miRNA or RBPs interacting with the target mRNA at that region, resulting in changes in mRNA expression. In addition, we hypothesize that the direct interaction of circRNA and mRNA results in double-stranded RNA hybrids, which might modulate RNA turnover processes, leading to changes in target mRNA expression.

Although CLiPPR-seq identified novel mRNA-interacting circRNAs, this study has a few limitations. Since the computational tools identify circRNAs based on the backsplice junction sequence, higher sequencing depth may lead to identification of more number of mRNA-interacting circRNAs (48). As circRNA identification tools use different algorithms for backsplice junction identification, a different circRNA annotation tool may identify a different subset of circRNAs (48,49). Furthermore, the exact region of circRNA–mRNA interactions was predicted computationally, which need to be validated experimentally by biochemical assays. Since circRNAs and mRNAs are known to associate with RBPs and miRNAs, the accessibility of the interacting sequence to interact with the target mRNA needs experimental validation. The effect of *in vivo* secondary and tertiary structure of the interacting RNAs in the presence of endogenous RBPs and miRNAs must be considered while analyzing the functional relevance of circRNA–mRNA hybrids. In addition, the exact mechanism of target mRNA expression regulation by interacting circRNAs needs further investigation.

Together, CLiPPR-seq not only identified novel interactions of hundreds of circRNAs with mRNAs but also indicated the necessity for studying circRNA-mRNA interactions deeply. We expect that the future application of CLiPPR-seq in mapping the global circRNA–mRNA interactome involving diverse organisms, a wide variety of cell/tissue types, developmental stages, and different disease conditions will help better understand the complex regulations through circRNA–mRNA interactions that currently lack known functions.

## Supplementary Material

gkae058_Supplemental_Files

## Data Availability

All the data generated in this study are included in the main text or Supplementary data. The RNA-seq data generated in this study were deposited in the European Nucleotide Archive with Accession number PRJEB58914.
