# Supplementary material for: Global identification of mRNA-interacting circular RNAs by CLiPPR-Seq
Source: Nucleic Acids Res. Author manuscript; Available in PMC 2024 Apr 15. (PMC11014417; doi:10.1093/nar/gkae058)
Supplement: Supplementary material [file EMS194846-supplement-Supplementary_material.pdf]

Global Identification of mRNA-Interacting Circular RNAs by CLiPPR-Seq

Suman Singh[1,2,†], Sharmishtha Shyamal[1,†], Arundhati Das[1], Amaresh C. Panda[1,*]

[1] Institute of Life Sciences, Nalco Square, Bhubaneswar, Odisha-751023, India
[2] Regional Center for Biotechnology, Faridabad, Haryana-121001, India
[†] Contributed equally
* To whom correspondence should be addressed: Email: amaresh.panda@ils.res.in

SUPPLEMENTARY TABLES AND FIGURES

Supplementary Table S1: CircRNA annotation of βTC6 CLiPP-CLiPPR-seq
Supplementary Table S2: CircRNA annotation of C2C12 CLiPP-CLiPPR-seq
Supplementary Table S3: CircRNA annotation of HeLa CLiPP-CLiPPR-seq
Supplementary Table S4: Human circRNA LiftOver to Mouse
Supplementary Table S5: CircRNA-mRNA BLAST analysis in βTC6 cells
Supplementary Table S6: CircRNA-mRNA BLAST analysis in C2C12 cells
Supplementary Table S7: CircRNA-mRNA BLAST analysis in HeLa cells
Supplementary Table S8: Oligonucleotides used in this study

Supplementary Figure S1

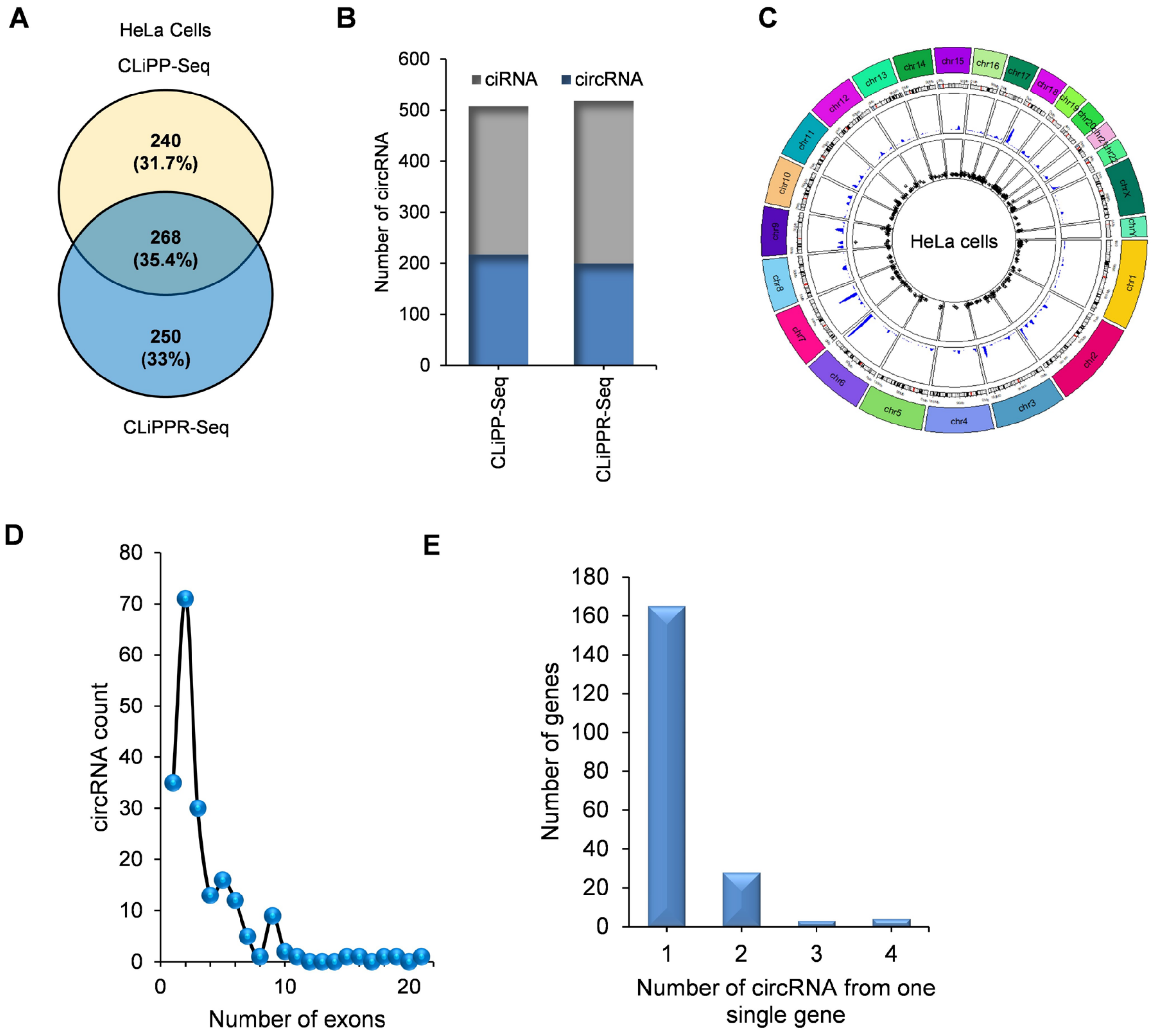

Supplementary Figure S1: Characteristics of circRNA-mRNA hybrids expressed in HeLa cells. A. CircRNAs identified by in HeLa cell CLiPPR seq and CLiPP-seq samples using CIRCexplorer2. **B**. The number of circRNAs that are generated from exonic (circRNA) or intronic (ciRNA) sequences in HeLa cells. **C.** Circos Plot showing the circRNAs on hg38 chromosomal axis for HeLa cells. The internal circle with black stars shows the distribution of length of the circRNAs. **D.** Dot plot showing the distribution of the number of exons in the circRNAs. **E.** Bar graph showing the number of genes and the number of circRNAs generated per gene.

Supplementary Figure S2

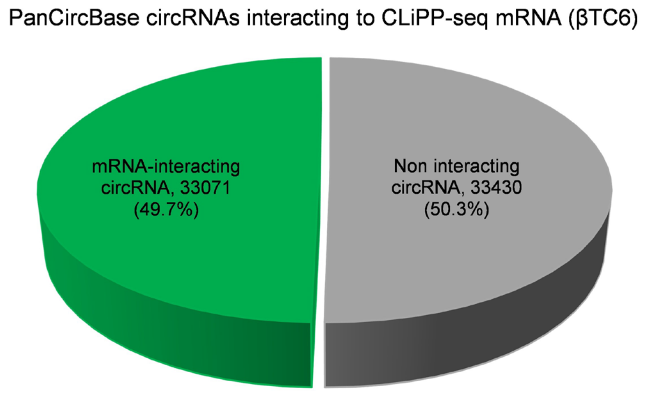

Supplementary Figure S2. BLAST analysis showing the number of interacting and non-interacting circRNAs in the PancircBase database with mRNAs expressed in βTC6 CLiPP-seq datasets.

Supplementary Figure S3

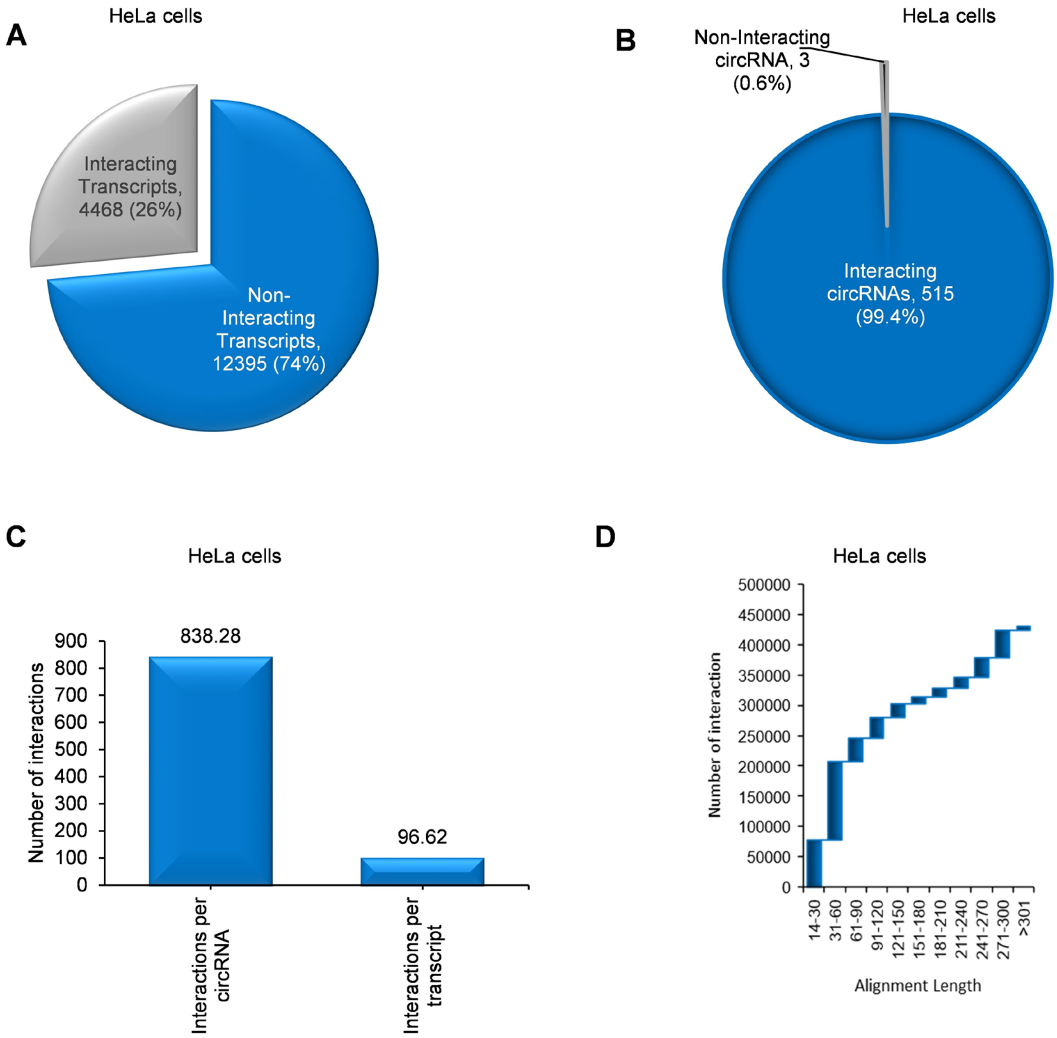

Supplementary Figure S3. BLAST analysis of circRNAs identified in CLiPPR-seq with mRNAs in CLiPP-Seq samples of HeLa cells. A. The number of mRNAs in HeLa cell CLiPP-seq interacting with circRNAs identified in CLiPPR-seq. **B.** Number of circRNAs in HeLa cell CLiPPR-seq samples with sequence complementarity with HeLa cell mRNAs. **C.** Graphs showing the number of interactions per mRNA and circRNA in HeLa cells. **D.** Graph shows the alignment length of circRNAs and the number of circRNA-mRNA hybrids in HeLa cells.

Supplementary Figure S4

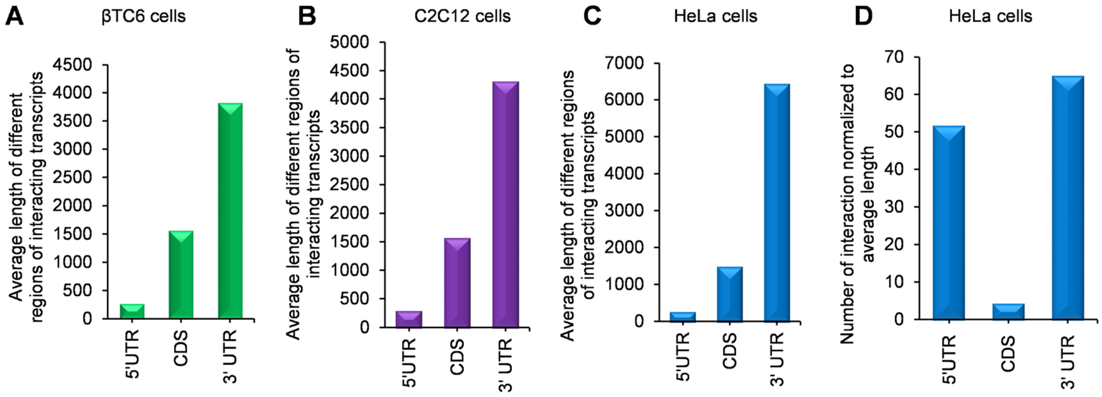

E

βTC6 cells				
**circRNA**	**circRNA_Chr ID (mm39)**		**Gene Name**	**Splice Length (nt)**
circAcbd3	chr1|180560730|180566202|+		Acbd3	617
circCdyl	chr13|35999891|36000558|+		Cdyl	667
circMtcl1	chr17|66743074|66745386|-		Mtcl1	384
circAsph	chr4|9635900|9639347|-		Asph	219
circEp400	chr5|110903262|110904632|-		Ep400	1370
circTnrc6a	chr7|122742972|122762100|+		Tnrc6a	494
circZfp609	chr9|65701903|65702777|-		Zfp609	874
**C2C12 cells**				
**circRNA**	**circRNA_Chr ID (mm39)**		**Gene Name**	**Splice Length (nt)**
circAcbd3	chr1|180560730|180566202|+		Acbd3	617
circMtcl1	chr17|66743074|66745386|-		Mtcl1	384
circEp400	chr5|110903262|110904632|-		Ep400	1370
circTnrc6a	chr7|122742972|122762100|+		Tnrc6a	494
circGse1	chr8|121280322|121280541|+		Gse1	219
circZfp609	chr9|65701903|65702777|-		Zfp609	874
**HeLa cells**				
**circRNA**	**circRNA_Chr ID (hg38)**	**mm39 liftOver_ID**	**Gene Name**	**Splice Length (nt)**
circGSE1	chr16|85633913|85634132|+	chr8|121280322|121280541|+	GSE1	219
circCDYL	chr6|4891712|4892379|+	chr13|35999891|36000558|+	CDYL	667
circASPH	chr8|61680967|61684188|-	chr4|9635900|9639347|-	ASPH	219

Supplementary Figure S4. A-C. Average length of 5' UTR, CDS, and 3' UTR of the interacting mRNAs in βTC6 (A), C2C12 (B), and HeLa (C) cells CLiPP-seq datasets. **D.** The bar graph shows the number of circRNA interactions normalized to the average length of different regions of interacting mRNAs in HeLa cells. **E.** List of circRNAs selected for validation in different cells.

Supplementary Figure S5

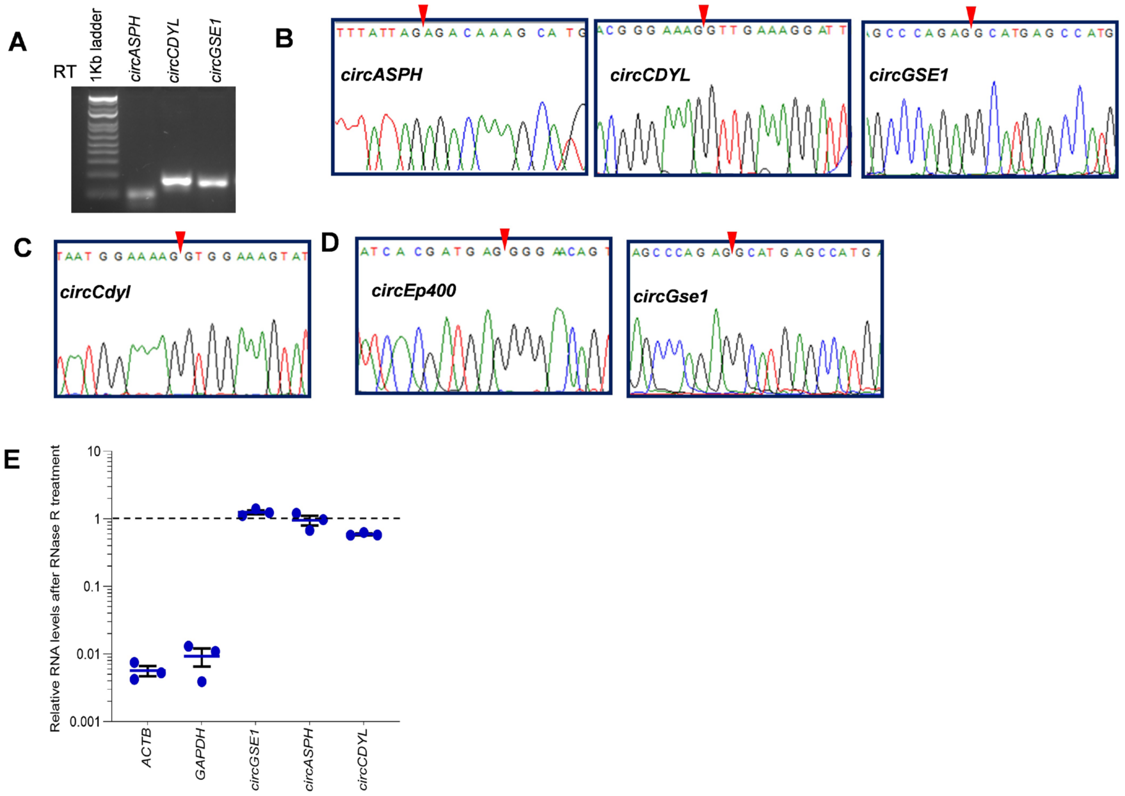

Supplementary Figure S5. Validation of selected mRNA-interacting circRNAs in HeLa cells. A. RT-PCR products of selected circRNAs in HeLa cells were resolved on a SYBR Gold-stained agarose gel. **B.** Sanger sequencing data shows the circRNA junction sequence of circRNAs in HeLa cells. **C-D.** Sanger sequencing data shows the circRNA junction sequence matching with that of RNA seq data in βTC6 (C) and C2C12 (D) cells. **E.** RT-qPCR analysis showing the levels of mRNAs and circRNAs upon RNase R treatment of total RNA from HeLa cells. The red arrowhead in panels B-D depicts the BSJ sequence.

Supplementary Figure S6

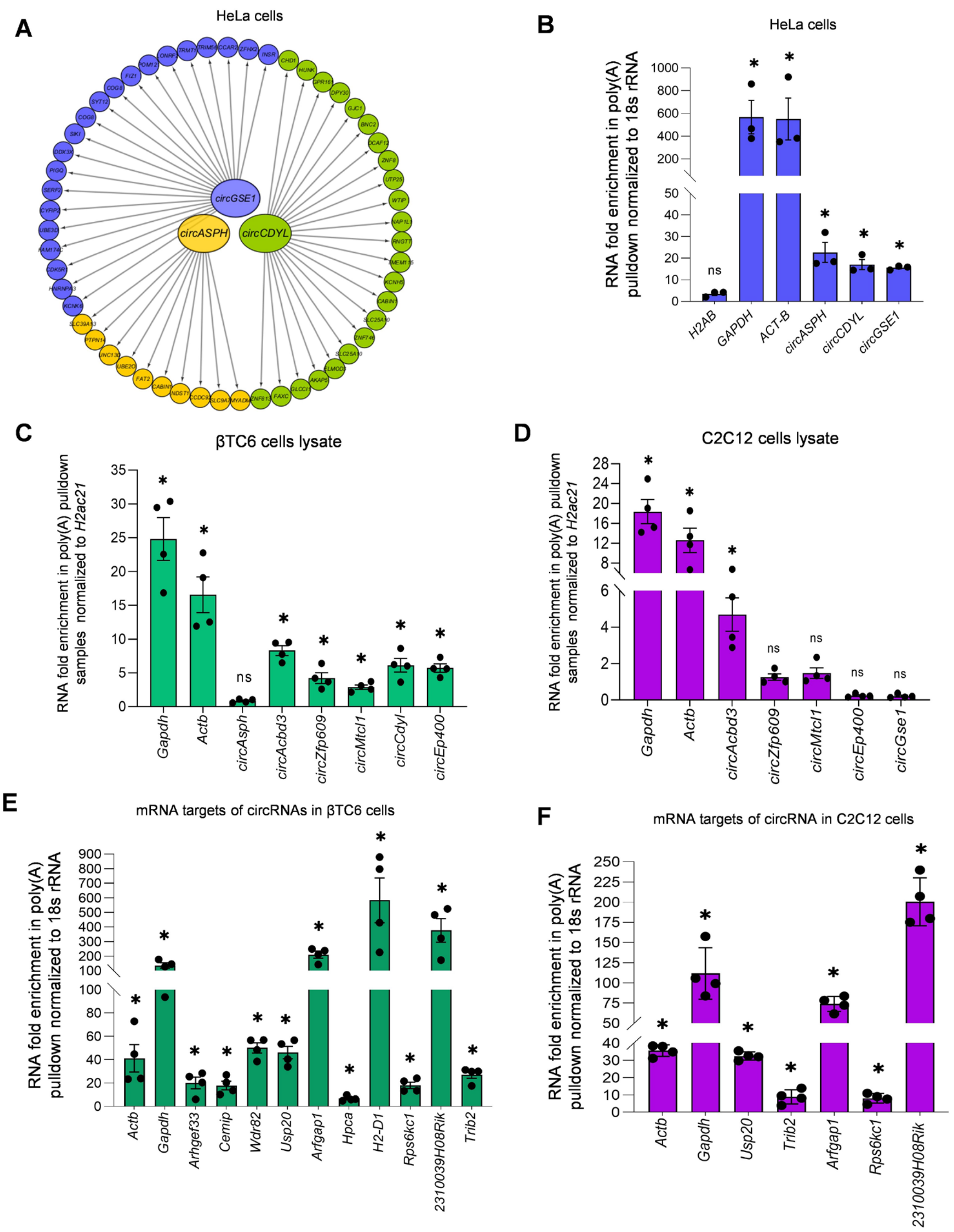

Supplementary Figure S6. A. Cytoscape image showing the mRNAs interacting with a subset of abundant circRNAs in HeLa cells. **B.** RT-qPCR analysis showing enrichment of circRNAs in poly(A) pulldown samples using HeLa cell total RNA compared to input. **C-D.** RT-qPCR analysis showing the enrichment of mRNAs and circRNAs in poly(A) pulldown samples compared to input using native cell lysates of βTC6 (C) and 2-day differentiated C2C12 cells (D). **E-F.** RT-qPCR analysis of mRNA in poly(A) pulldown samples showing the enrichment of circRNAs interacting with the mRNAs in βTC6 (E) and 2-day differentiated C2C12 cells (F) cells compared to input. The error bars in panel B-F represent means ± SEM from 3-4 independent experiments, and * indicates the statistical significance with p-value < 0.05.

Supplementary Figure S7

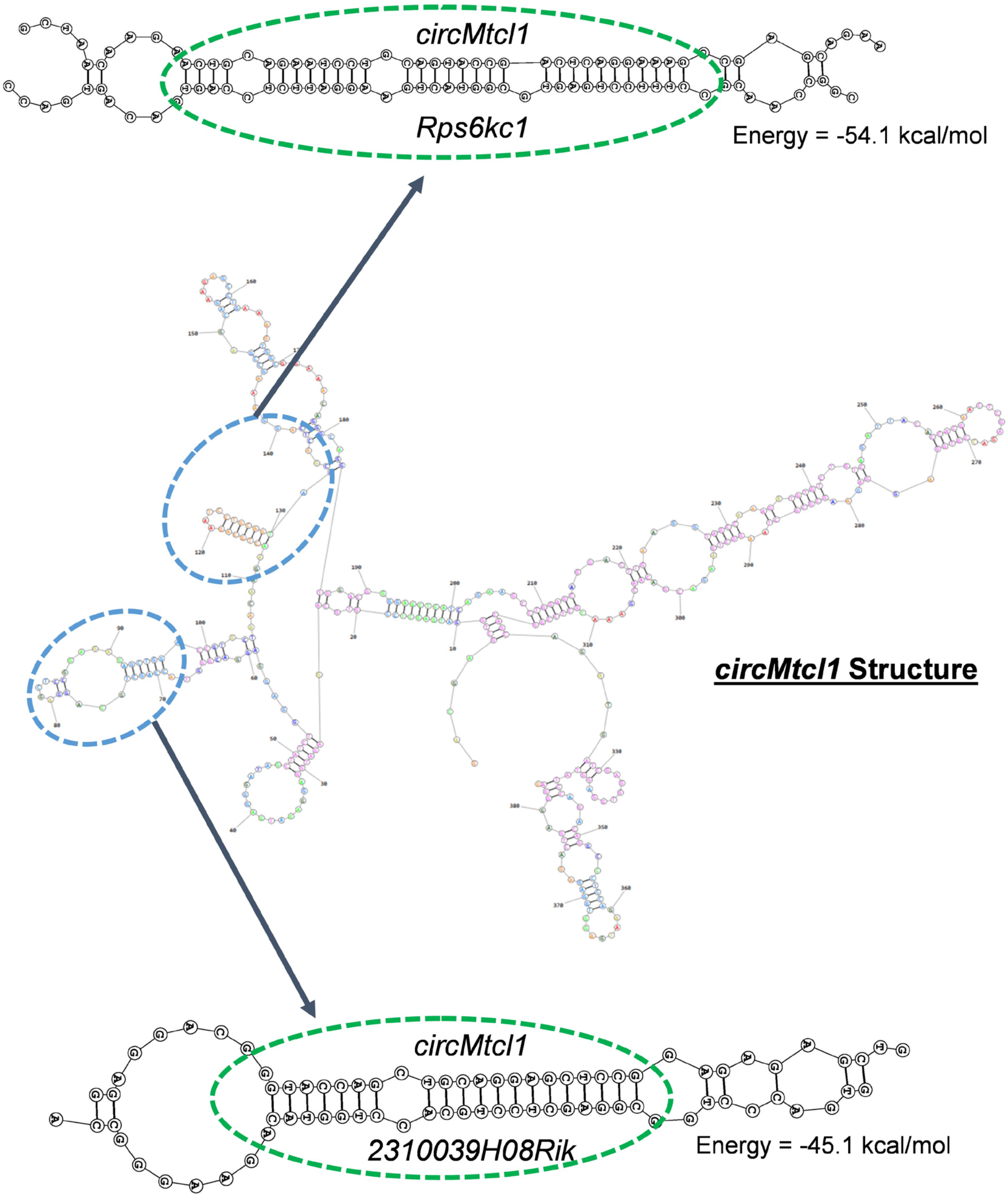

Supplementary Figure S7: Secondary structure of *circMtcl1* predicted by RNAstructure web server. The blue dotted circles indicate the region of interaction in *circMtcl1.* The green dotted circles indicate the bimolecular secondary structure and region of interaction of *circMtcl1* with the interacting sequences of target *2310039H08Rik* and *Rps6kc1* mRNA predicted by the DuplexFold algorithm of RNAstructure web server.

Supplementary Figure S8

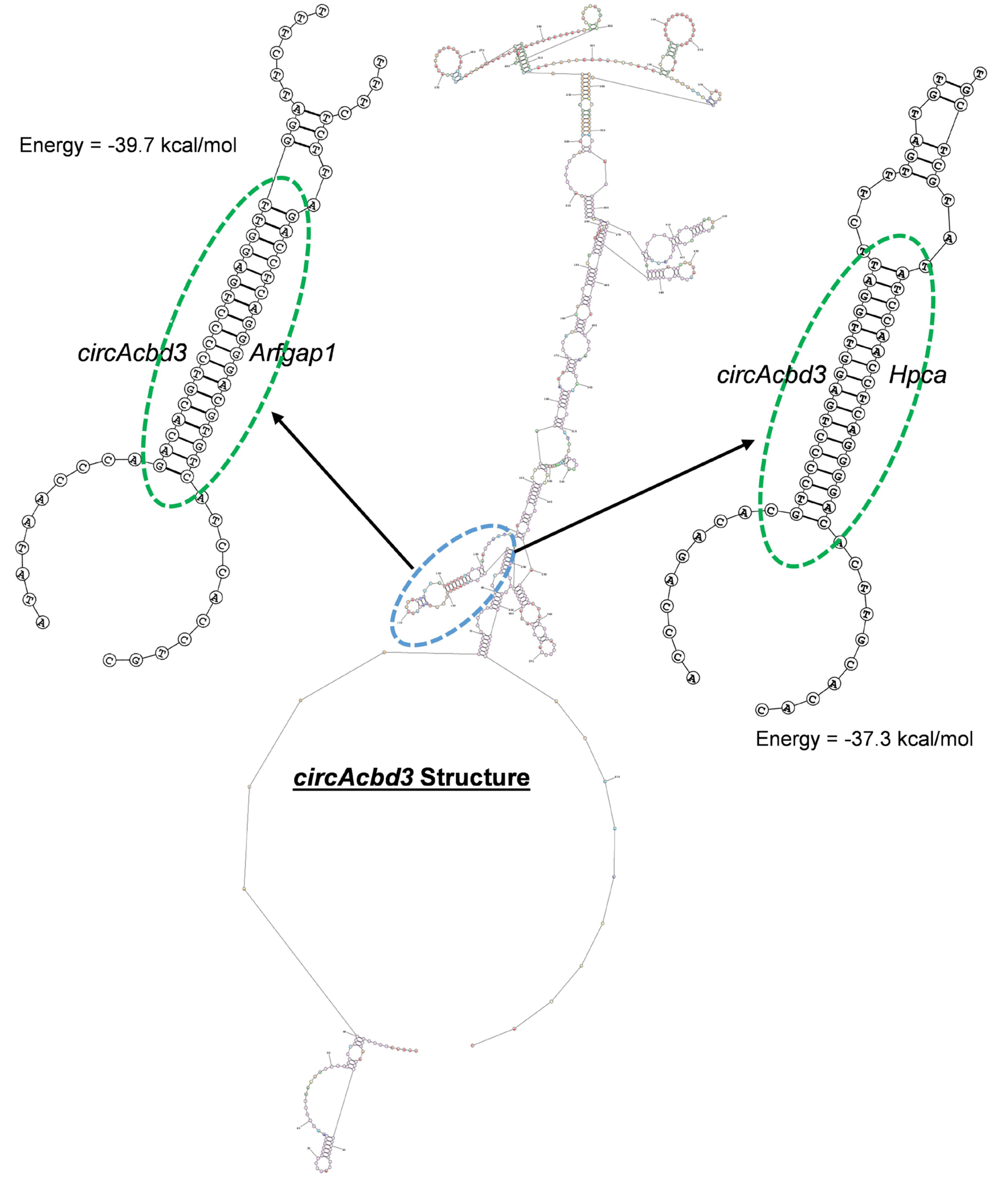

Supplementary Figure S8: Secondary structure of *circAcbd3* predicted by RNAstructure web server. The blue dotted circles indicate the region of interaction in *circAcbd3.* The green dotted circles indicate the bimolecular secondary structure and region of interaction of *circAcbd3* with the interacting sequences of target *Hpca* and *Arfgap1* mRNA predicted by the DuplexFold algorithm of RNAstructure web server.

Supplementary Figure S9

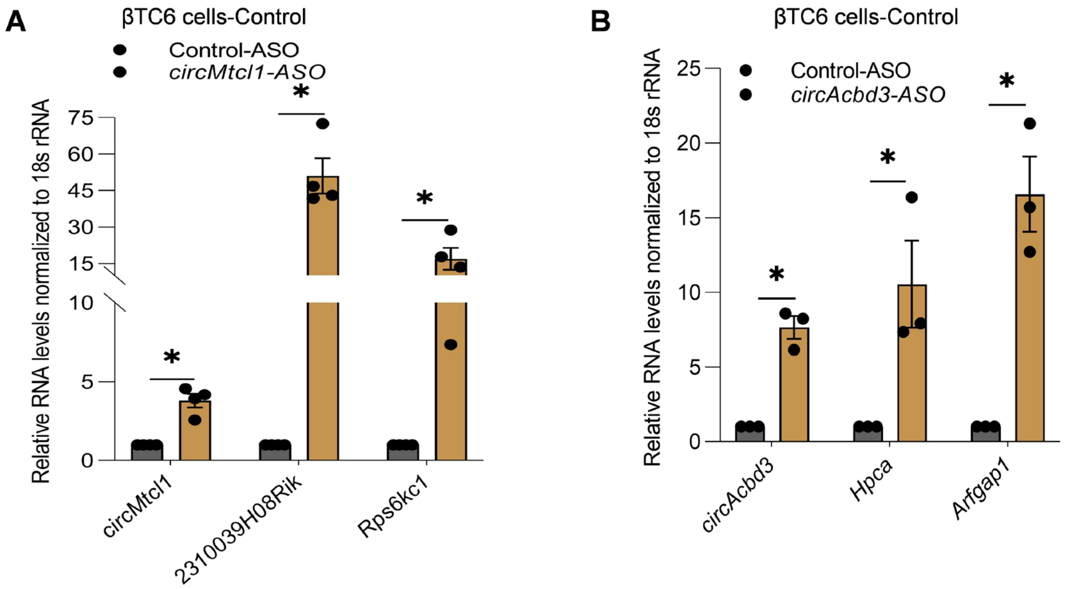

Supplementary Figure S9. Enrichment of target mRNAs in circRNA pulldown. A-B. RT-qPCR analysis shows the enrichment of circRNAs and their target mRNAs in *circMtcl1* (A) and *circAcbd3* (B) ASO pulldown samples compared to control ASO pulldown in βTC6 cells without AMT crosslinking. The error bars represent means ± SEM from 3 to 4 independent experiments, and * indicates the statistical significance with a p-value < 0.05.

Supplementary Figure S10

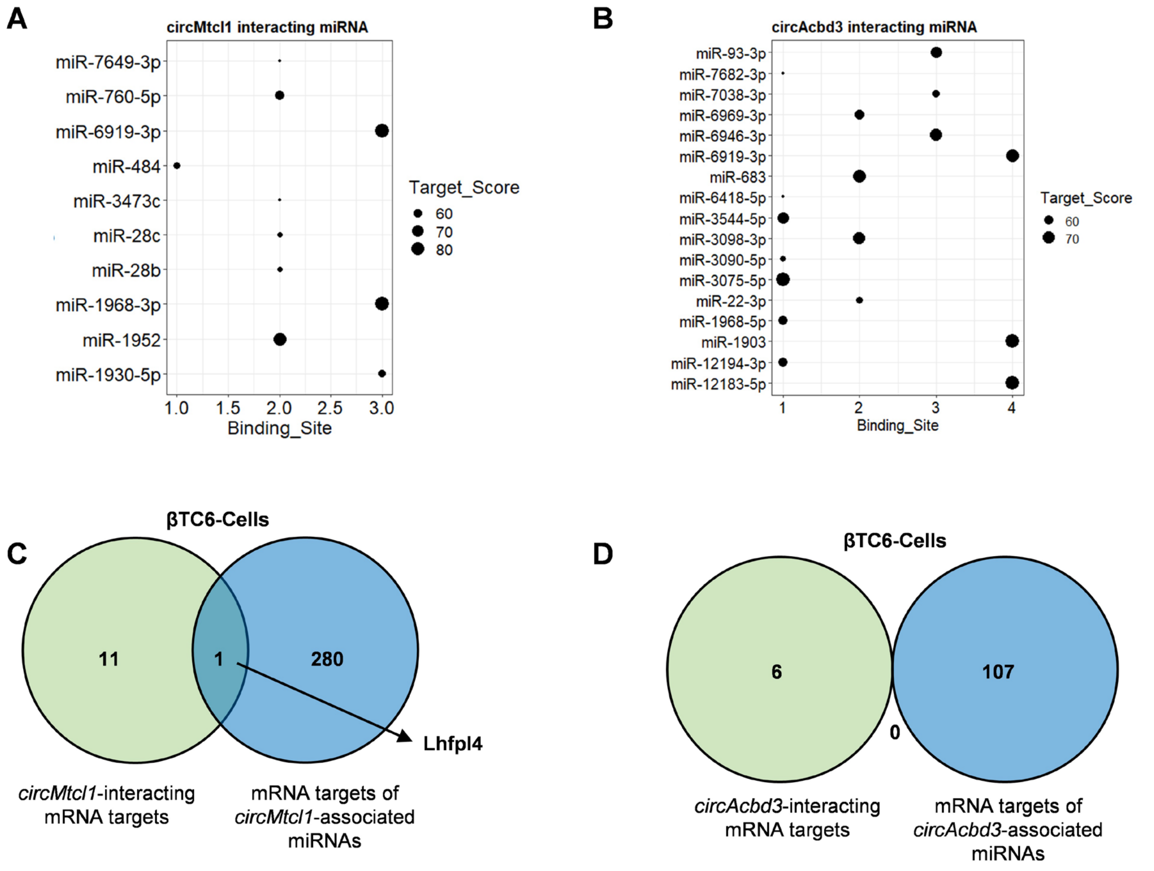

Supplementary Figure S10. miRNA regulatory axis of *circMtcl1* and *circAcbd3*. A-B. Dot plot showing the number of binding sites and target scores of miRNAs predicted by miRDB to interact with *circMtcl1* (A) and *circAcbd3* (B). **C.** Venn diagram showing the miRTarBase-predicted mRNA targets of miRNAs associated with *circMtcl1* and the mRNA targets of *circMtcl1* identified by BLAST in βTC6 cells. **D.** Venn diagram showing the miRTarBase-predicted mRNA targets of miRNAs associated with *circAcbd3* and the mRNA targets of *circAcbd3* identified by BLAST in βTC6 cells.
